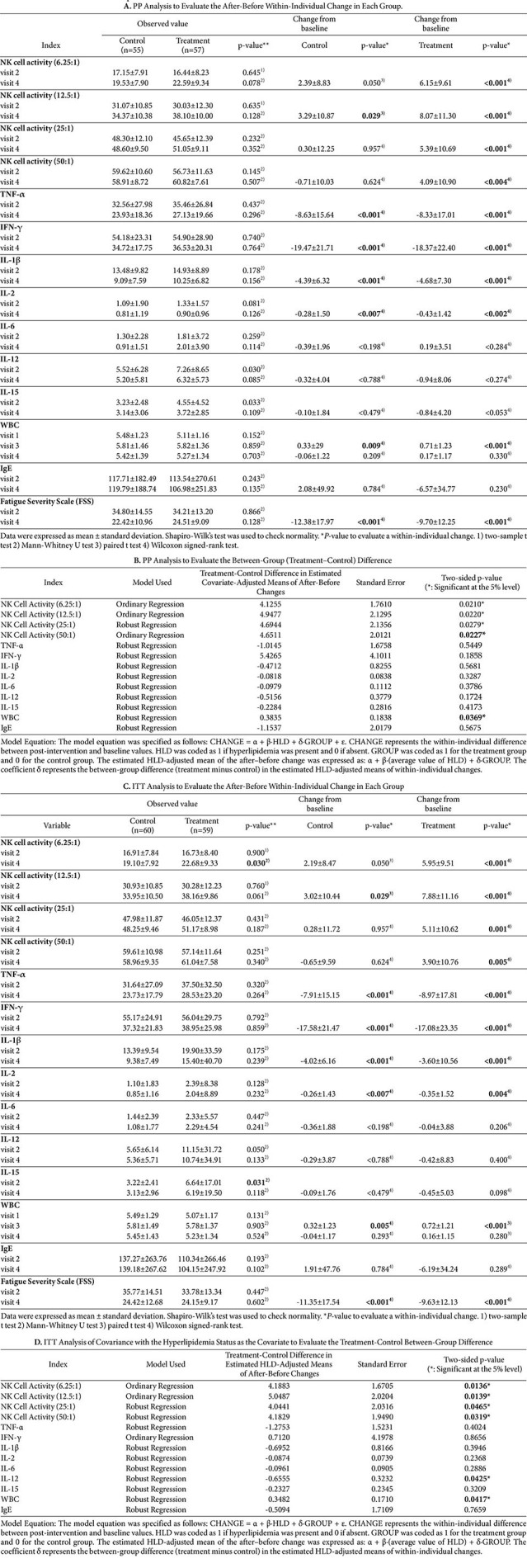# Microbiome-Modulating Effects of Heat-Treated *Lactiplantibacillus plantarum* LM1004 and Its Enhancement of NK Cell Activity: Evidence from a Clinical Trial and a Simulated Human Intestinal Microbiome Ecosystem

**DOI:** 10.4014/jmb.2604.04046

**Published:** 2026-07-03

**Authors:** Sukyung Kim, Hoonhee Seo, Tae-Rahk Kim, Md Abdur Rahim, Hanieh Tajdozian, Youjin Yoon, Sujin Jo, Md Sarower Hossen Shuvo, Indrajeet Barman, Chaeeun Park, Saebim Lee, Sangwon Lee, Min Hyung Cho, Jangho Ha, Hansol Hong, Sohee An, SungJune Chu, Minn Sohn, Yoo Kyung Ahn, Yoon Ju So, Sungsue Rheem, Seong-Ho Han, Ho-Yeon Song

**Affiliations:** 1 LactoMason Co., Ltd., 13-10 Worasan-ro 950 beon-gil, Gyeongnam 52840, Republic of Korea; 2 Department of Microbiology and Immunology, School of Medicine, Soonchunhyang University, Chungnam 31151, Republic of Korea; 3 K-Microbiome Institute, Soonchunhyang University, Chungnam 31538, Republic of Korea; 4 Department of Family Medicine, College of Medicine, Dong-A University, Busan 49201, Republic of Korea; 5 Bioinformatics and Molecular Design Research Center (BMDRC), 209, Veritas A Hall, Yonsei University, Incheon 21983, Republic of Korea; 6 Division of Big Data Science, Korea University, Sejong-si 30019, Republic of Korea

**Keywords:** Heat-treated *Lactiplantibacillus plantarum* LM1004, Simulated human intestinal microbiome ecosystem, Low-abundance beneficial bacteria, Natural killer cell activity, Microbiome-based functional food and therapeutics

## Abstract

Probiotics are increasingly recognized for their capacity to modulate gut microbiota, regulate microbial metabolic activity, and influence host immune responses, thereby contributing to the maintenance of immune homeostasis and overall health. In this study, we assessed the efficacy and safety of heat-treated *Lactiplantibacillus plantarum* LM1004 (HT-LM1004) in a randomized, placebo-controlled clinical trial and explored its mechanisms of action in a simulated human intestinal microbiome ecosystem. After 8 weeks of supplementation, we observed significantly enhanced natural killer (NK) cell activity with a concurrent improvement in white blood cell (WBC) counts relative to the placebo group, suggesting an overall enhancement of the host's primary immune defense baseline within the normal physiological range. Mechanistic investigations within the simulated human intestinal microbiome ecosystem demonstrated that HT-LM1004 increased microbial species diversity in the ascending colon (AC), followed by elevated richness in the transverse colon (TC) and descending colon (DC) at the End and Post time points, suggesting selective enrichment of low-abundance beneficial bacterial taxa. Metabolomics analyses indicated compartment-specific changes, especially within bile acid metabolism pathways, while non–bile acid metabolites were predominantly enriched in the DC. Short-chain fatty acid (SCFA) profiling also revealed distinct, time-dependent changes across the different gut compartments. Collectively, these results indicate that *L. plantarum* LM1004 boosts NK cell activity in humans by enriching low-abundance beneficial bacteria and modulating their metabolic products, underscoring its promise as a microbiome-based functional food and preventative option to support immune health.

## Introduction

The gut microbiota is now recognized as a pivotal regulator of host physiological processes, influencing immune system maturation, metabolic homeostasis, and predisposition to disease. Disruptions in microbial diversity and metabolite profiles have been linked to increased risk of infections, chronic inflammation, and metabolic disorders [1, 2]. Consequently, interventions aimed at restoring ecosystem balance and enhancing the production of immunoactive metabolites—notably bile acids and short-chain fatty acids (SCFAs)—are increasingly regarded as critical approaches for promoting host immune resilience [3, 4].

Probiotics have traditionally been investigated as functional ingredients with the potential to enhance immune function. In contrast to probiotics, which are defined as live microorganisms, postbiotics—such as the heat-treated HT-LM1004 used in this study—refer to a 'preparation of inanimate microorganisms and/or their components that confers a health benefit on the host'. Despite this distinction, their effects have frequently been assessed using proxy endpoints, including cytokine fluctuations or activation of natural killer (NK) cells, often without consideration of the ecological and metabolic intricacies of the gut microbiome. To facilitate advancement from being perceived solely as “immune-enhancing supplements” to being recognized as microbiome-based therapeutics, it is crucial to situate probiotic effects within the context of host–microbiome interactions, applying systems-level methodologies such as metagenomics and metabolomics [5, 6]. These analytical platforms facilitate the detection of low-abundance keystone taxa and enable the delineation of immunomodulatory metabolites that can modulate immune effector mechanisms, offering critical mechanistic insights that connect microbial community restructuring to systemic immune responses.

*Lactiplantibacillus plantarum* LM1004 has recently gained recognition as a notable postbiotic strain in this context. Prior research has suggested that HT-LM1004 mitigates cyclophosphamide-induced immunosuppression in *in vivo* [7], activates host immune signaling via TLR2/MAPK/NF-κB pathways in *in vivo* [8], and displays immunostimulatory effects in *in vitro* macrophage assays [9]. Furthermore, recent findings from a randomized, double-masked clinical trial indicate that oral administration of HT-LM1004 modulates innate immunity in human subjects [10]. Nevertheless, while these investigations have established a valuable foundation, they have primarily focused on targeted immunological responses, such as cytokine regulation, macrophage activation, and host immune signaling pathways, without comprehensively evaluating ecosystem-level interactions such as gut microbiota composition, microbial functional dynamics, and metabolomic alterations.

This study was specifically designed to address previous limitations by integrating a randomized, placebo-controlled clinical trial with a spatially resolved human intestinal microbiome simulator. The clinical trial allowed rigorous evaluation of whether HT-LM1004 supplementation improves immune function in healthy adults, while the *in vitro* ecosystem enabled detailed monitoring of microbial diversity, detection of rare taxa, and compartment-specific metabolite changes [11, 12]. By aligning data from human efficacy trials with mechanistic insights obtained through metagenomic and metabolomic analyses, this study moves beyond mere descriptive correlations to formulate a coherent mechanistic basis for HT-LM1004. This integrative research strategy demonstrates how probiotics can be advanced as microbiome-based therapeutics, connecting gut microbiota restructuring with clinically meaningful immunological outcomes.

## Methods

### Production and Preparation of Heat-Treated *L. plantarum* LM1004

Heat-treated *L. plantarum* LM1004 (KCCM 43246) was manufactured by Lactomason Co., Ltd. (Republic of Korea), using sequential laboratory- and pilot-scale processes adapted from previously established protocols outlined in the company’s patents and publications [7, 8]. The *L. plantarum* LM1004 strain, originally isolated from kimchi, was taxonomically identified by 16S rRNA gene sequencing, and its strain-specific characteristics have been described previously [9]. The production process involved controlled fermentation, followed by biomass recovery, heat treatment, and drying. Accordingly, the final material is a heat-inactivated, dried bacterial cell preparation derived from a fermented culture. The product primarily consists of non-viable bacterial cells and their structural components formed during bacterial growth prior to heat treatment. Viability testing was conducted to confirm complete inactivation, and the results met the established criteria for an inactivated microbial preparation. In this study, HT-LM1004 was provided by the Department of Production, Lactomason Co., Ltd. The material was formulated as freeze-dried powder, produced under tightly controlled conditions and subjected to validated quality-control protocols at the company’s GMP-compliant production facility. The heat treatment was applied after fermentation and biomass recovery to ensure complete microbial inactivation while preserving the structural components of the inactivated bacterial cells. Structural components include the cell wall and cell envelope, such as peptidoglycan, lipoteichoic acids, surface proteins, and other cell-associated molecules that form during bacterial growth. These components serve as key microbe-associated molecular patterns (MAMPs) that are recognized by host pattern recognition receptors (PRRs), such as Toll-like receptor 2 (TLR2), thereby initiating innate immune signaling pathways. Such preserved cellular structures may contribute to the observed immune-related effects, without implying the presence of viable bacteria or isolated metabolites. For strict traceability, batch 20OTF8 was assigned to the preparation used in the clinical trial, while batch 10PTF6 was assigned to the *in vitro* intestinal fermentation model. Specifying separate batch identifiers ensures transparency and reproducibility in documenting material provenance and aids in the precise interpretation and replication of study findings across multiple experimental platforms.

### Design and Methodology of the HT-LM1004 Clinical Trial

This study was an 8-week, randomized, double-blind, placebo-controlled, single-center clinical trial at Dong-A University Hospital (Busan, Republic of Korea) to assess the efficacy and safety of HT-LM1004 in enhancing immune function among healthy adults. This randomized, double-blind, placebo-controlled clinical trial was conducted in accordance with the Declaration of Helsinki and the Ethical Guidelines for Clinical Research. The reporting of this trial adheres to the Consolidated Standards of Reporting Trials (CONSORT) guidelines, and the completed CONSORT checklist has been provided as a supplementary file. The study protocol was approved by the Institutional Review Board of Dong-A University Hospital (IRB no. DAUHIRB-23-152), and all participants provided written informed consent before enrollment., in accordance with the [13]. This study also registered at ClinicalTrials.gov (NCT07433855). Eligible participants were healthy men and women aged from 19 to 75 years meeting the following criteria: (1) Perceived Stress Scale (PSS) score ≥16 at screening, (2) a peripheral white blood cell (WBC) count between 3 × 10^3^ and 10 × 10^3^ cells/μL, (3) a documented history of ≥2 episodes of upper respiratory tract infection within the previous year, and (4) willingness to adhere to all study procedures. Major exclusion criteria comprised body mass index ≥30 kg/m^2^, recent vaccination within 2 months, COVID-19 infection within the previous 2 months, history of systemic diseases with potential immune system involvement, use of immunomodulatory medication or probiotics within 4 weeks, current pregnancy or lactation, and allergy to any component of the investigational product. These specific inclusion criteria were established to recruit individuals with a relatively compromised immune baseline or heightened susceptibility to physiological immune fluctuations, allowing for a more sensitive evaluation of the immunomodulatory potential of HT-LM1004. In total, 120 participants were assigned in a 1:1 ratio to receive either HT-LM1004 or a placebo. Randomization was conducted using a computer-generated sequence, and allocation was concealed by using identical-looking supplement containers. Both subjects and investigators remained blinded to group allocation throughout the trial.

The investigational product consisted of a capsule containing 500 mg/day of HT-LM1004 powder, manufactured under Good Manufacturing Practices. The active capsules did not contain additional active ingredients or neutral fillers other than the HT-LM1004 material itself. The product consists primarily of heat-inactivated bacterial cells recovered after fermentation and subsequent thermal processing. In contrast, the placebo capsules contained maltodextrin and were matched to the active product in appearance, weight, and packaging. Participants were instructed to ingest one capsule per day following dinner for 8 weeks. The placebo contained maltodextrin and matched the active product in appearance, weight, and packaging. Compliance was monitored at each scheduled visit by counting remaining capsules, and subjects with compliance below 80% were excluded from the per-protocol analysis.

The primary endpoint was the change in NK cell activity from baseline to week 8. NK cell cytotoxicity was evaluated using a 51Cr-release assay with K562 target cells, following previously established protocols [14]. Peripheral blood mononuclear cells (PBMCs) were isolated from whole blood using Lymphoprep™ (StemCell) and then NK cells were isolated from the isolated PBMC by using Mojosort™ Human NK cell isolation Kit (BioLegend, USA). NK cell activity was assessed at effector-to-target (E:T) ratios of 6.25:1, 12.5:1, and 25:1. Secondary endpoints included measurements of serum cytokine levels (TNF-α, IFN-γ, IL-1β, IL-2, IL-6, IL-12, IL-15), total WBC count, total IgE concentrations, and PSS scores. Cytokine concentrations including TNF-α, IFN-γ, IL-1β, IL-2, IL-6, IL-12, and IL-15, were determined by a multiplex bead-based immunoassay [15]. These specific cytokines were selected to comprehensively assess the immunomodulatory effects of HT-LM1004, focusing on markers essential for NK cell development and activation (IL-12 and IL-15), effector function (IFN-γ), and the regulation of the systemic inflammatory environment (TNF-α, IL-1β, and IL-6). WBC counts and differentials were measured using an automated hematology analyzer, and total IgE concentrations were determined using a chemiluminescent immunoassay [16]. The PSS questionnaire, validated for use in Korean populations, was utilized to assess perceived stress [17]. The PSS was used exclusively as a screening tool to confirm eligibility at enrollment (Table S1). Safety assessment comprised the evaluation of adverse events (AEs), clinical laboratory parameters (hematology and blood chemistry), and vital signs at baseline and week 8, in accordance with Good Clinical Practice guidelines [18].

Statistical analyses were conducted using SPSS version 26 (Windows version 26.0, Armonk, NY, IBM Corp.) and SAS^®^ software version 9.4 (SAS Institute Inc., USA). Continuous variables were reported as mean ± standard deviation (SD), while categorical variables were reported as frequencies and percentages. Efficacy analyses were performed on both the intention-to-treat (ITT) and per-protocol (PP) populations. The ITT population comprised all randomized participants who ingested the study product at least once and provided at least one post-baseline efficacy measurement. In contrast, the PP population consisted of individuals with no major protocol deviations and compliance rates ≥80%. Within-group comparisons from baseline to week 8 were evaluated using paired t-tests for normally distributed variables or Wilcoxon signed-rank tests for non-normally distributed variables. Between-group differences were assessed using analysis of covariance (ANCOVA), with baseline values as covariates to control for potential confounders [19]. All tests were two-sided, and a *p*-value < 0.05 indicated statistical significance. Missing data in the ITT analysis were handled using the last observation carried forward (LOCF) approach. Safety assessments covered all participants who received at least one dose of the study product, with adverse events summarized descriptively by group.

### Simulated Human Intestinal Microbiome Ecosystem Setup and Experimental Procedure

A twin-unit Simulated Human Intestinal Microbiome Ecosystem (SHIME^®^ system, ProDigest BVBA, Belgium) was utilized to replicate the physicochemical and microbiological environments of the human colon *in vitro* [20]. Each system consisted of vessels simulating the ascending colon (AC), transverse colon (TC), and descending colon (DC), all maintained with continuous pH regulation, temperature control at 37°C, and regular anaerobic gas flushing. A standardized nutrient medium (PDNM001B; consisting of arabinogalactan, pectin, xylan, starch, glucose, yeast extract, peptone, mucin, and L-cysteine HCl), pancreatic juice solution (including NaHCO_3_, pancreatin, and oxgall), and anaerobic buffer were continuously supplied, with the nutrient medium and pancreatic juice prepared in 3 L batches and replenished once or twice weekly. Acid and base solutions were refreshed as necessary to sustain the target pH in each vessel. The fecal inoculum was sourced from healthy male and female donors in a 1:1 ratio was purchased from BioBankHealing Co., Ltd. (Republic of Korea), homogenized under anaerobic conditions, then aliquoted and stored at -80°C until used. Prior to seeding the microbial community, thawed fecal slurry was added to the AC, TC, and DC compartments at 5% (v/v) of their working volumes. The experiment was structured into three phases: a 1-week stabilization, a 4-week probiotic intervention, and a 1.5−week washout phase. HT-LM1004, provided as a freeze-dried powder, was stored at room temperature until needed. For each daily administration (0.1 g, corresponding to 2 × 10^10^ cells), the powder was suspended in 10 mL sterile distilled water and delivered solely to treatment unit at 10:30 a.m., five times per week over four weeks. Sampling occurred at four distinct time points: before administration (July 22), during administration (August 5), at the conclusion of administration (August 19), and after administration (August 28). At each time point, 10 mL of fermentation broth was withdrawn from each of the AC, TC, and DC; the first 5 mL was discarded and the next 5 mL collected into sterile tubes (three tubes per compartment). Samples were immediately frozen at −80°C for subsequent 16S rRNA gene sequencing, short-chain fatty acid determination, and targeted microbial analyses.

### Metagenomic Analysis of 16S rRNA Gene Amplicons Derived from Simulated Human Gut Samples

This 16S rRNA gene-based metagenomic analysis was conducted in accordance with our previous investigations [21-24]. Total metagenomic DNA was isolated from the collected samples using the QIAamp DNA Mini Kit (Qiagen, Germany), following the manufacturer’s protocol. All instruments and work surfaces were decontaminated with 70% ethanol before use, and sterile filter tips were consistently used to reduce the risk of external contamination. Each sample underwent homogenization, treatment for protein digestion and cell wall disruption, and DNA was eluted in nuclease-free water. DNA yield was quantified using a Qubit 4 fluorometer (Thermo Fisher Scientific, UK), and sample integrity was verified by electrophoresis on 0.8% agarose gels. Purity was assessed through measurement of the A260/A280 ratio, and DNA samples were preserved at -20°C until downstream processing. Negative controls lacking template were incorporated at each step to monitor for potential contamination, and quality control parameters of DNA integrity, amount, and purity were documented. For library preparation, the V4 hypervariable region of the 16S rRNA gene was amplified utilizing primers that included Illumina Nextera XT overhang adapter sequences (Forward: 5′-TCGTCGGCA GCGTCAGATGTGTATAAGAGACAGCCTACGGGNGGCWGCAG-3′; Reverse: 5′-GTCTCGTGGGCTCGGAGATGTGTATAAGAGACA GGACTACHVGGGTATCTAATCC-3′), specifically targeting the 515–806 bp region with a predicted amplicon length of 359 bp. PCR amplification was performed with 2× KAPA HiFi HotStart ReadyMix (Kapa Biosystems, USA) and carried out according to the manufacturer’s recommended cycling parameters. Following amplification, amplicons were purified with AMPure XP beads (Beckman Coulter, UK) to eliminate residual primers and other PCR reagents. Purified PCR products underwent an additional indexing PCR step using the Nextera XT DNA Library Prep Kit (Illumina, USA) to incorporate dual indices and sequencing adapters for library multiplexing. The indexed libraries were again purified using AMPure XP beads, and quantification was performed with a Qubit fluorometer; purity was further confirmed using a NanoDrop spectrophotometer. DNA concentrations were then normalized to ensure all libraries were pooled at equimolar ratios, resulting in a single combined library. To enhance sequence diversity, 10% PhiX Control library (Illumina) was incorporated into the pooled libraries. The prepared library mixture was denatured before being loaded onto the Illumina iSeq100 platform for paired-end sequencing (2 × 300 bp). Sequencing run quality control included instrument calibration and assessment of the proportion of base calls with quality scores ≥Q30. Primary sequence reads were processed with a QIIME 2-based pipeline following our previous published protocols [25], which encompassed demultiplexing, stringent quality filtration, error correction, and taxonomic classification. After performing rarefaction to achieve a consistent sequencing depth, alpha diversity metrics (Shannon index, Faith’s phylogenetic diversity, and observed features) as well as beta diversity (weighted UniFrac) were calculated, and differences in microbial community structure between groups were assessed.

### Short-Chain Fatty Acid Quantification in Simulated Intestinal Fermentation Samples

Short-chain fatty acids (SCFAs) present in effluents from an *in vitro* human gut microbiome fermentation system were quantified using headspace gas chromatography–mass spectrometry (GC–MS). For each analysis, 1 mL of effluent was combined with 2.5 g of sodium chloride (NaCl) and 1 mL of 2% sulfuric acid in a sealed headspace vial. The samples were equilibrated in a TurboMatrix Headspace Sampler (PerkinElmer, USA), then introduced into a PerkinElmer Clarus 690 GC equipped with a Clarus SQ8 MS detector and an Elite-FFAP capillary column (30 m × 0.25 mm i.d., 0.25 μm film thickness). The injector was maintained at 250°C and delivered 0.16 mL in split mode (5:1); helium served as the carrier gas at a flow rate of 16 mL/min. The oven temperature was programmed to increase from 120°C to 200 °C at 5°C/min, and the ion source was set to 250°C, operating in full-scan mode. Calibration curves were generated using standard solutions (1–100 mg/L) for acetic, propionic, butyric, and valeric acids. SCFA identification in the samples was achieved by comparing the retention times and mass spectra with those of authentic standards.

### Metabolomics Analysis

All LC–MS/MS data collection, metabolite classification, and statistical analyses were conducted at the Bioinformatics and Molecular Design Research Center (BMDRC, Republic of Korea). Metabolomic profiling utilized LC–MS/MS, and only features with confirmed MS/MS spectra (*i.e.*, fragmentation patterns verified upon energy application) were included as reliable metabolites. Culture broth samples (300 μL) were collected at different time points from the Ascending Colon (AC), Transverse Colon (TC), and Descending Colon (DC) compartments of the Simulator of the Human Intestinal Microbial Ecosystem (SHIME) after inoculation with heat-treated *Lactiplantibacillus plantarum* LM1004. For metabolite extraction, 200 μL of a pre-chilled (-20°C) solvent, consisting of a 1:3 (v/v) mixture of acetonitrile and methanol, was added to each sample. The resulting mixture was vortexed thoroughly and subsequently centrifuged at 14,000 rpm for 10 min at 4°C. Following centrifugation, the supernatant was collected and filtered through a 0.22 μm Polyethersulfone (PES) syringe filter. The final filtrate was used for Ultra-High Performance Liquid Chromatography-Tandem Mass Spectrometry (UHPLC-MS/MS) analysis.

The final filtrate was analyzed by ultra-high-performance liquid chromatography coupled to high-resolution tandem mass spectrometry using an Orbitrap Exploris 120 mass spectrometer. UHPLC separation was performed using a Thermo Vanquish Horizon UHPLC system equipped with a Hypersil GOLD™ Vanquish C18 column (2.1 × 150 mm, 1.9 μm, 175 Å). The column temperature was maintained at 40°C, the autosampler temperature at 4°C, the flow rate at 0.3 mL/min, and the injection volume was 1 μL. Mobile phase A consisted of 0.1% formic acid in water, and mobile phase B consisted of 0.1% formic acid in acetonitrile. The gradient elution program was set as follows: 5% B from 0 to 3 min, 11.5% B at 7 min, 12% B at 8 min, 14% B at 15 min, 25% B at 25 min, 50% B from 27 to 29 min, 60% B at 34 min, 100% B from 36 to 50 min, and re-equilibration to 5% B from 55 to 60 min.

Mass spectrometric data were acquired using a Full MS–data-dependent MS/MS (Full MS–ddMS^2^) method in both positive and negative electrospray ionization modes. The MS1 and MS/MS resolutions were set to 120,000 and 15,000, respectively, with a scan range of m/z 140–1,400. Higher-energy collisional dissociation (HCD) was applied using assisted collision energies of 30, 40, and 50. The ion transfer tube temperature and vaporizer temperature were set to 320°C and 300°C, respectively. The sheath gas and auxiliary gas were set to 50 and 10, respectively. The electrospray voltage was set to 3,500 V in positive ion mode and 2,500 V in negative ion mode. All samples were analyzed in triplicate, and the resulting peak area values were averaged for subsequent metabolite abundance analysis.

The relative abundance of each metabolite was expressed as the ratio of the LC–MS/MS signal intensity to the total ion chromatogram (TIC) of each sample, thereby normalizing signal intensities across all samples. For quantification, the detected peak area values (area_control_n, area_exp_n) were used after subtracting signals derived from the blank, with abundances expressed relative to the sample's TIC. While minor discrepancies may occur between chromatographic quantification and MS peak area, these proxy values were adopted for metabolite abundance and validated through internal correlation analysis. Two complementary cheminformatics resources were applied for metabolite classification: NPClassifier (NPC), which focuses on natural products, and ClassyFire, a hierarchical framework for chemical taxonomy. Since NPC has been reported to expand the natural product classification capacity of ClassyFire, the results from NPC were given primary consideration. Only classification outputs with a probability ≥ 0.8 were deemed reliable. For statistical testing, within-subject (time: baseline, week 2, week 4, and follow-up after 1.5 weeks post-intervention) as well as between-subject variables (control versus experimental groups) were incorporated. Consequently, a mixed-design analysis of variance (Mixed ANOVA) was applied to assess metabolite variation. Significant findings were extracted when both within- and between-subject factors achieved statistical significance (*p* < 0.05) and showed a substantial effect on variance in the dependent variables, as reflected by high partial eta squared (ηp^2^) values. Statistically significant metabolic alterations were further illustrated using line and bar charts (all_metabolites_pattern.png), facilitating qualitative evaluation of relative abundance across the experimental conditions (AC, TC, DC) and groups. Although statistical analyses highlighted significant changes, readers should interpret the graphical results in a comprehensive manner, as visual representations may not always align precisely with statistical outcomes.

## Results

### Efficacy Outcomes of HT-LM1004 Supplementation

**Fig. 1:**
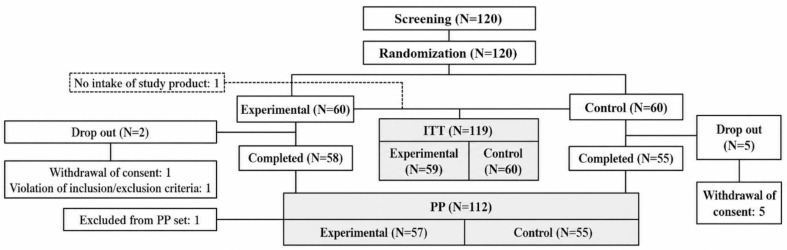
Flow diagram of participant screening, randomization, and analysis populations (ITT and PP). Eligible participants were randomly assigned in a 1:1 ratio to either the HT-LM1004 (treatment) group or the placebo (control) group and followed for 8 weeks. The intention-to-treat (ITT) population included all randomized participants who received at least one dose of the investigational product and had at least one post-baseline assessment. The per-protocol (PP) population consisted of participants who completed the study without being excluded due to consent withdrawal, major protocol violations, or poor compliance. Group comparisons presented in the tables refer to the treatment (HT-LM1004) and control (placebo) groups within each analysis population.

The diagram of participant progression through the study, covering screening, randomization, and inclusion in analysis sets, is illustrated in Fig. 1. A total of 120 individuals were evaluated and randomly allocated to either the experimental group (HT-LM1004, n = 60) or the placebo group (n = 60). After accounting for withdrawals and exclusions, 59 participants from the experimental group and 60 from the control group were included in the intention-to-treat (ITT) analysis. In comparison, the per-protocol (PP) analysis comprised 57 and 55 participants, respectively. Compliance in this trial was specified as consuming at least 80% of the intended dosage. All participants in both the ITT and PP groups met the compliance criterion of greater than 80%, demonstrating strong adherence to the supplementation protocol (Table S2). For the ITT analysis, mean (± standard deviation) compliance rates for visits 2–3, 3–4, and 2–4 were 97.75 ± 4.45%, 99.04 ± 2.39%, and 98.60 ± 2.70% in the control group, and 97.74 ± 4.16%, 98.32 ± 3.97%, and 97.93 ± 3.48% in the experimental group. No significant differences in compliance rates were detected between groups (*p* = 0.479, 0.487, and 0.157). The PP analysis revealed compliance rates of 98.25 ± 3.65%, 99.04 ± 2.39%, and 98.60 ± 2.70% in the control group, and 97.66 ± 4.21%, 98.29 ± 4.00%, and 97.90 ± 3.50% in the experimental group, also without significant between-group differences (p = 0.212, 0.460, and 0.138). Baseline demographic and clinical parameters of the participants are summarized (Table S3). Both groups had a mean age in the mid-40s and were comparable with respect to sex distribution. There were no substantial differences in height, weight, body mass index, or blood pressure between the groups. Lifestyle variables such as smoking status and alcohol use also did not differ significantly. Most initial laboratory test results and vital sign measurements fell within standard reference ranges. A minor, statistically significant difference in baseline body temperature was observed in the experimental group during PP analysis (*p* < 0.05); however, this finding lacked clinical significance. Despite comparable baseline characteristics across groups, HT-LM1004 supplementation resulted in significant improvements in immune-related outcomes, most notably the primary endpoint of NK cell activity (at all E:T ratios) and secondary endpoints including total WBC counts and IL-12 levels. Specifically, NK cell activity, the primary endpoint, showed consistent increases across all effector-to-target (E:T) ratios in the HT-LM1004 group compared with the placebo group in both ITT and PP analyses. In addition, selected immune-related biomarkers demonstrated favorable trends in the treatment group. These findings indicate that HT-LM1004 supplementation enhanced innate immune function in healthy adults under stress conditions. Based on these clinical observations, further *in vitro* studies were conducted to explore potential mechanistic pathways underlying the observed immunomodulatory effects.

In the PP population, NK cell activity, the primary outcome, assessed at effector-to-target (E:T) ratios of 6.25:1, 12.5:1, 25:1, and 50:1, showed pronounced increases in the HT-LM1004 group, whereas the control group exhibited minimal changes. Also, WBC, as a secondary outcome related to the immune-related biomarkers, showed a statistically significant increase in this group (Table 1A and 1B). Similar trends were detected in the ITT population, with the HT-LM1004 group displaying consistent elevations across all E:T ratios. Also, some of the secondary outcomes, including immune-related biomarkers such as IL-12 and WBC counts, showed statistically significant differences between groups throughout the intervention period (Table 1C and D). Given the substantial inter-individual variability observed in cytokine responses, these secondary outcomes should be interpreted with caution and are presented as supportive findings rather than primary efficacy measures (Fig. S1).

Blood lipid parameters are summarized (Table S4). In the PP population, the mean values of total cholesterol, triglycerides, HDL cholesterol, and LDL cholesterol did not differ noticeably between the HT-LM1004 and control groups at any visit. Assessment of within-group changes from baseline revealed a modest numerical reduction in total cholesterol and triglycerides in both groups, whereas HDL and LDL cholesterol levels remained largely unchanged. The ITT population displayed a comparable trend, with no statistically significant differences between groups for any lipid parameter at any visit. These results suggest that HT-LM1004 supplementation had no negative impact on lipid metabolism throughout the 8-week intervention. Baseline medical history by organ system is presented (Table S4). In both ITT and PP populations, the prevalence of pre-existing conditions—including dermatologic, musculoskeletal, ophthalmologic, otologic, gastrointestinal, hepatic, metabolic/endocrine, cardiovascular, respiratory, hematologic, immune, neurologic, or surgical histories—remained low and was similarly balanced across groups. But the experimental group in the ITT analysis showed a slightly higher frequency of hyperlipidemia (*p* = 0.049), it was selected as a covariate for ANCOVA. Collectively, the baseline comorbidity distribution supports that randomization produced clinically comparable groups. Concomitant medication use during the study period was documented (Table S5), and detailed information on individual medications and their indications is provided in Table S6, which lists the specific medications used by each participant and their corresponding clinical indications. According to Table S5, in the ITT populationnearly three-quarters of participants in both groups reported using at least one medication, while the remainder reported no medication use. The majority of these medications were related to chronic conditions not associated with the study endpoints. The pattern was similar in the PP population, with no significant group differences observed in either the proportion of participants taking medications or the types of medications used. These results further indicate that concomitant treatment use was well balanced between groups throughout the trial.

### Safety Profile of HT-LM1004 Based on Adverse event Monitoring, Laboratory Tests, and Vital Sign Measurements

Adverse events observed during the study are summarized (Table S7). Only mild disorders of the respiratory system were reported, which consisted exclusively of typical cold-related symptoms such as cough and rhinorrhea. These adverse events occurred in 8.47% of participants in the HT-LM1004 group (n = 5) and 6.67% of participants in the control group (n = 4), and no moderate or severe events were reported in either group. Cases of pneumonia were not observed in the HT-LM1004 group; in contrast, a single case (1.67%) was reported in the control group. Every adverse event was transient, resolved without residual effects, and was deemed not related to the study product. Hematological and biochemical results are provided (Table S8). The majority of measured values remained within normal physiological parameters for the duration of the study, and there were no clinically significant differences between the groups. In the ITT analysis, certain parameters, including hemoglobin, hematocrit, RBC, and select liver function tests (ALT, AST), showed statistically significant changes from baseline within groups; these changes were minor, not linked to clinical symptoms, and did not yield significant differences between groups. Lipid profiles, electrolytes, renal indices, and inflammatory markers (CRP, ESR) remained stable in both groups during the 8-week trial. The recorded vital signs—including body temperature, blood pressure, and pulse—are shown (Table S9). Measurements remained consistent across visits for both groups, and no significant differences between groups were identified at any assessment time point. While minor within-group variations in body temperature and systolic blood pressure occurred in the control group, these changes were not deemed clinically significant. Medical history assessed using questionnaires (Table S10) did not indicate any new or worsening conditions attributable to the intervention. Symptom and diagnosis prevalence were similar between groups, and there were no indications of increased risk associated with HT-LM1004. Taken together, supplementation with HT-LM1004 for 8 weeks was well tolerated, without evidence of adverse events related to treatment or clinically relevant changes in laboratory measures, vital signs, or reported medical history.

### Establishment and Operational Overview of the Simulated Human Intestinal Microbiome Ecosystem for HT-LM1004 Administration

**Fig. 2:**
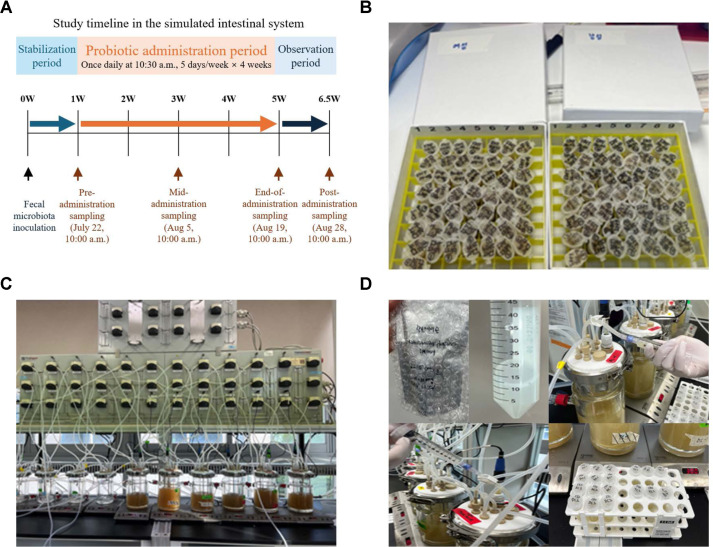
Simulated Human Intestinal Microbiome Ecosystem experiment assessing the microbi-ome-modulating properties of HT-LM1004. A twin-unit Simulated Human Intestinal Microbiome Ecosystem was utilized to investigate how HT-LM1004 influences microbiome composition. In the experimental setup, one unit was maintained as an untreated control, while the parallel unit received HT-LM1004 treatment. (**A**) The experimental design included a 1-week period for system stabilization, a 4-week probiotic treatment phase, and a 1.5-week observational phase thereafter. (**B**) Fecal samples were sourced from healthy male and female donors at a 1:1 ratio and (**C**) were introduced into ascending colon (AC), transverse colon (TC), and descending colon (DC) vessels at a 5% volume proportion of each jar bottle. (**D**) HT-LM1004 was administered daily as 2 × 10^10^ cells (0.1 g freeze-dried powder in 10 mL distilled water) into AC2 at 10:30 a.m., five days weekly for the 4-week intervention period according to protocol. Sampling of 5 mL was carried out pre-administration, at the midpoint and end of administration, as well as after administration, all at 10:00 a.m., with subsequent storage at -80°C for later microbiome profiling.

The experimental setup for assessing the microbiome-modulating properties of HT-LM1004 using a simulated human intestinal microbiome ecosystem is illustrated (Fig. 2). The study design included a 1-week stabilization phase, a 4-week probiotic intervention, and a 1.5-week post-intervention observation (Fig. 2A). Sampling was systematically performed at four predetermined points: pre-administration (July 22), mid-administration (August 5), end-of-administration (August 19), and post-administration (August 28), with samples collected at 10:00 a.m., ahead of each daily probiotic dose. Fecal inocula were generated using equal contributions from healthy male and female donors, homogenized anaerobically, and partitioned for use in the study (Fig. 2B). This mixture was dispensed into the ascending colon (AC), transverse colon (TC), and descending colon (DC) vessels of the simulation model at a volume comprising 5% of each vessel’s working capacity (Fig. 2C). Maintenance of the system involved continuous regulation of pH and temperature, together with anaerobic gas flushing, to ensure physiological conditions. HT-LM1004 was prepared daily from freeze-dried powder (0.1 g, representing 2 × 10^10^ cells, in 10 mL sterile distilled water), and administered exclusively into the AC2 vessel at 10:30 a.m., five times weekly for four weeks (Fig. 2D). Sample collection was conducted before each administration to ensure that analyses of the microbiome captured the cumulative effects rather than immediate post-dosing responses. For each time point, 5 mL of fermentation broth from every colon compartment was stored at –80°C pending downstream microbiome and metabolic analyses. This arrangement enabled accurate tracking of temporal shifts in microbial community structure and metabolic activity throughout HT-LM1004 exposure and following its withdrawal, while rigorously maintaining experimental and environmental controls.

### Effects of HT-LM1004 on Microbial Community Diversity in the Human Intestinal Microbiome Simulator

**Fig. 3:**
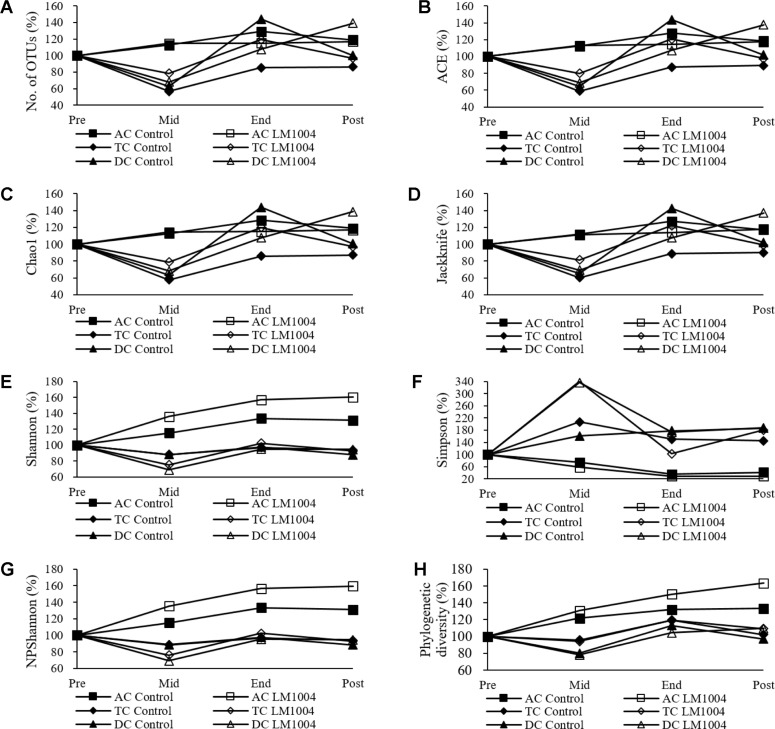
Impact of probiotic supplementation on microbial community diversity within the human intestinal microbiome simulator. Alpha diversity indices of the gut microbiota were measured in the ascending colon (AC), transverse colon (TC), and descending colon (DC) compartments at baseline (Pre), Mid, End (after 4 weeks of supplementation), and Post (after 1.5 weeks of withdrawal), with results expressed as percentage changes from baseline. (**A**) The number of operational taxonomic units (OTUs, (**B**) the ACE index, (**C**) the Chao1 index, and (**D**) the Jackknife index serve as indicators of species richness. No significant differences in richness were found in the AC during the intervention, whereas an increase was noted in the TC at the End point and in the DC at the Post point. (**E**) The Shannon index, (**F**) the Simpson index, and (**G**) the NPShannon index reflect the evenness of community composition, while (**H**) phylogenetic diversity represents the evolutionary relationships among microbial communities. In these assessments, the TC and DC showed no notable changes, while the AC exhibited a sustained increase throughout all evaluated time points.

Alpha diversity metrics of the gut microbiota were determined in the AC, TC, and DC vessels at baseline (Pre), Mid, End (after 4 weeks of supplementation), and Post (1.5 weeks following withdrawal), and described as percentage changes from baseline (Fig. 3).

Regarding richness estimators, the number of OTUs, the ACE index, the Chao1 index, and the Jackknife index exhibited trends unique to each compartment (Fig. 3A-3D). No significant changes were found in the AC throughout the study, while a notable increase in richness was observed in the TC at the End time point and in the DC at the Post time point. These data indicate that HT-LM1004 supplementation selectively facilitated the proliferation of low-abundance bacterial taxa in more distal compartments, rather than causing uniform shifts throughout the gut. Indices of species diversity, as measured by the Shannon, Simpson, NPShannon, and phylogenetic diversity metrics, showed a consistent increase in the AC but did not vary significantly over time in the TC and DC (Fig. 3E−3H). In summary, these results suggest that HT-LM1004 supplementation initially altered species diversity within the proximal colon, followed by subsequent increases in richness and evenness predominantly in downstream compartments.

### Effects of 4-Week HT-LM1004 Supplementation on Gut Microbial Composition

**Fig. 4:**
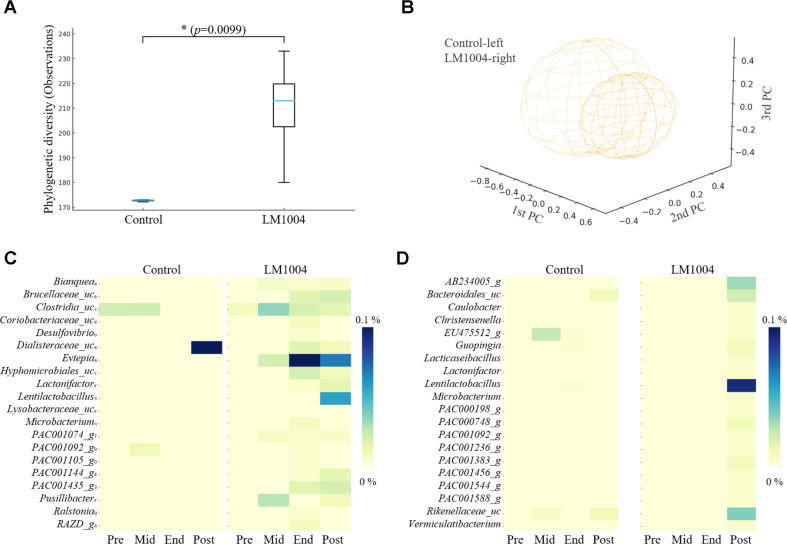
Effects of HT-LM1004 supplementation on gut microbial composition and diversity. To further elucidate the previously observed increased species diversity in the AC and enhanced richness in the TC and DC, a detailed analysis was conducted. At the endpoint (after 4 weeks of supplementation), (**A**) phylogenetic diversity in the AC was significantly higher in the probiotic group than in the control group (*p = 0.0099). (**B**) Principal coordinate analysis (PCoA) of β-diversity revealed a distinct separation of microbial communities. Additionally, β-set significance analysis was conducted showing statistical significance with *p < 0.05. Heatmaps for the (**C**) TC and (**D**) DC compartments display the relative abundance of bacterial genera and operational taxonomic units (OTUs), demonstrating marked differences in distribution between the groups.

To further characterize the diversity changes in the AC and the increased richness in the TC and DC, additional analyses were conducted (Fig. 4). At the End time point (after 4 weeks of supplementation), the AC displayed significantly greater phylogenetic diversity in the LM1004- supplemented group versus controls (*p* = 0.0099) (Fig. 4A). Principal coordinate analysis (PCoA) of β-diversity clearly distinguished the microbial communities of the control and HT-LM1004 groups (Fig. 4B). Heatmap analyses in both the TC and DC revealed marked differences in the temporal distribution of bacterial genera and OTUs between groups across all sampled time points (Pre, Mid, End, and Post) (Fig. 4C and 4D). Importantly, HT-LM1004 supplementation was linked to the targeted enrichment of low-abundance taxa, including certain genera with previously reported health-associated properties. These alterations became apparent in the TC during supplementation and persisted to some degree after cessation, whereas in the DC, enrichment tended to intensify at the Post time point. Collectively, these findings indicate that the effects of HT-LM1004 supplementation on community structure extended beyond the period of intervention.

### Differential Metabolite Profiles in the Intestinal Compartments Following HT-LM1004 Supplementation

An untargeted metabolomics approach was employed. The resulting LC–MS/MS spectra from each environmental and temporal replicate underwent a data processing pipeline that included peak picking, alignment, and gap filling to extract quantifiable features reflecting temporal changes. This process yielded 1,688 (positive) and 1,199 (negative) features in the AC; 1,139 (positive) and 679 (negative) in the TC; and 1,135 (positive) and 662 (negative) in the DC. For metabolite identification, these features were first matched against authentic standard libraries. Remaining unidentified features were then annotated using the CANOPUS tool to predict their chemical class.

**Fig. 5:**
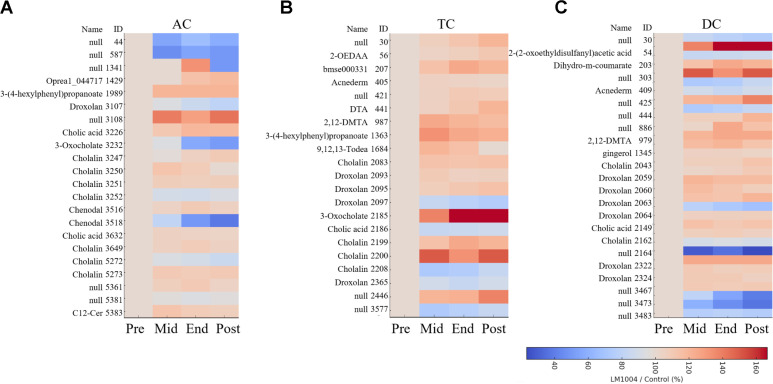
Distinct metabolite profiles in intestinal compartments after HT-LM1004 supplementation. Heatmaps display the relative abundance of metabolites detected in the ascending colon (AC), transverse colon (TC), and descending colon (DC) at baseline (Pre), mid-intervention (Mid), end of intervention (End), and post-intervention (Post). LC–MS/MS–based metabolomics analysis using mixed-design ANOVA demonstrated significant, compartment-specific alterations in metabolite profiles between the control and probiotic groups. (**A**) In the AC, probiotic supplementation led to a relative increase in primary bile acids, including cholic acid and chenodeoxycholic acid. In contrast, certain cholate derivatives, including cholalin and chenodal 3518 (Chenodeoxycholic acid), exhibited a decrease. (**B**) In the TC, more substantial metabolic shifts were present, notably a marked increase in several secondary bile acids, including deoxycholic acid (droxolan), 3-oxocholate, cholalin, 2,12-DMTA, OEDAA, and DTA. In contrast, signals corresponding to some primary bile acids declined. (**C**) In the DC, metabolic changes extended beyond bile acids: several secondary bile acids (*e.g.*, droxolan, cholic acid derivatives) were elevated, and specific non–bile acid metabolites, such as 3-(4-hexylphenyl) propanoate and Oprea1_044717, were also upregulated, indicating more comprehensive metabolic remodeling. C12-Cer, (2S)-2-[[7-[[(1S)-1-carboxy-5-(dodecanoylamino)pentyl]amino]-7-oxoheptanoyl]amino]-6-(dodecanoylamino)hexanoic acid <Lauroyl-ceramide derivative>; 2-OEDAA, 2-(2-oxoethyldisulfanyl)acetic acid <Thioacetic acid derivative>; DTA, Acetic acid, 2,2''-[carbonylbis(thio)]bis-<Bis(thiocarboxymethyl) ketone>; 2,12-DMTA, 2,12-dimethylidenetridecanedioic acid; null, not identified.

LC–MS/MS–based metabolomics employing mixed-design ANOVA revealed significant compartment-specific alterations in metabolite profiles between the HT-LM1004 and control groups (Fig. 5). In the AC, HT-LM1004 supplementation exhibited an increased abundance of primary bile acids, such as cholic acids(3226 and3632) and a chenodeoxycholic acid(3516), while several cholalin derivatives and a chenodeoxycholic acid(3518). Additional metabolites unrelated to bile acids, including 3-(4-hexylphenyl)propanoate and Opreal_044717, also showed elevated levels, indicating region-specific metabolic adaptations (Fig. 5A). In the TC, more pronounced shifts were observed, featuring notable increases in multiple secondary bile acids, including 3-oxocholate, cholalin, 2,12-DMTA (dimethyl taurocholic acid), OEDAA (oxoethyl deoxycholic acid amide), and DTA (deoxytaurocholic acid), as well as a relative reduction in primary bile acid signals. Enrichment of non–bile acid metabolites such as 3-(4-hexylphenyl)propanoate further supported evidence of broader metabolic perturbations (Fig. 5B). Changes in the DC extended beyond bile acid metabolism. Secondary bile acids, including various droxolan derivatives, exhibited a pronounced increase, whereas primary bile acids (*e.g.*, cholic acid) remained relatively unchanged. Additionally, levels of non–bile acid metabolites, such as 2-(2-oxoethyldisulfanyl)acetic acid, dihydro-m-coumarate, and gingerol, were elevated, along with persistent alterations in cholalin derivatives (Fig. 5C). The observed shift in the primary to secondary bile acid ratio, particularly the increase of deoxycholic acid (droxolan) in the descending colon, indicates a substantial alteration of the gut metabolic environment. Secondary bile acids are known to be potent signaling molecules that modulate host immune responses, often through receptors like the farnesoid X receptor (FXR) [1, 2]. They play a crucial role in maintaining gut homeostasis by promoting the differentiation of anti-inflammatory regulatory T cells (Tregs) and inhibiting pro-inflammatory T helper 17 (Th17) cells [3, 4]. Therefore, the HT-LM1004-induced increase in secondary bile acids suggests an enhanced potential for creating a more tolerogenic immune environment in the gut mucosa. In summary, these results indicate that HT-LM1004 supplementation drives compartment-specific metabolic changes, with bile acid pathway remodeling predominating in the AC and TC, and a more extensive range of bile acid and non–bile acid metabolites affected in the DC. The compartment-restricted alterations imply that LM1004 mediates distinct, site-dependent effects on both microbial and host-metabolite interactions.

### Effects of HT-LM1004 Supplementation on Short-Chain Fatty Acid Levels

**Fig. 6:**
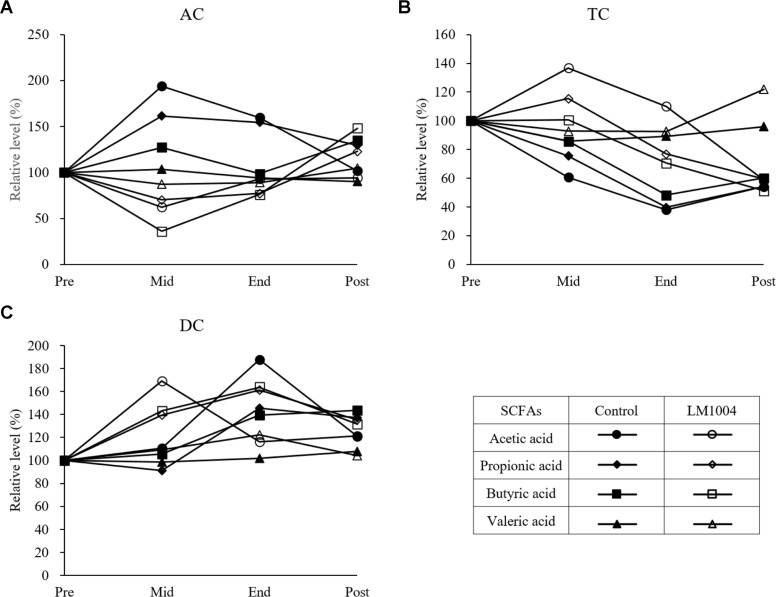
Effects of probiotic supplementation on short-chain fatty acid (SCFA) concentrations across intestinal compartments. The influence of probiotic supplementation on SCFA concentrations was evaluated, with results expressed as percentage change from baseline. Four SCFAs—acetic acid, propionic acid, butyric acid, and valeric acid—were quantified in the ascending colon (AC), transverse colon (TC), and descending colon (DC) at four time points: baseline (Pre), mid-intervention (Mid), end of intervention (End, 4 weeks), and post-intervention (Post, 1.5 weeks after cessation). (**A**) In the AC, SCFA concentrations declined at the midpoint but recovered to similar or higher levels at the End and Post time points. (**B**) In the TC and (**C**) DC, SCFA concentrations increased at the Mid and End points, showing a trend toward baseline values at the Post time point.

The effect of HT-LM1004 supplementation on short-chain fatty acid (SCFA) concentrations was evaluated in the AC, TC, and DC compartments, with results presented as percentage changes from baseline (Fig. 6). Four SCFAs—acetic acid, propionic acid, butyric acid, and valeric acid—were quantified at baseline (Pre), mid-intervention (Mid), end of intervention (End, 4 weeks), and post-intervention (Post, 1.5 weeks following cessation). In the AC, a decline in SCFA levels was observed at Mid, which subsequently rebounded to baseline or higher concentrations at the End and Post time points (Fig. 6A). In the TC, SCFA concentrations increased during the Mid and End periods and then trended back toward baseline after discontinuation (Fig. 6B). In the DC, this temporal trend was mirrored, with elevations at the Mid and End points followed by a partial return to baseline after intervention cessation (Fig. 6C). Together, these data demonstrate that HT-LM1004 supplementation induces dynamic and time-dependent modulation of SCFA production, with distinct regional responses characterized by temporary reductions in the AC and enrichment within the TC and DC during supplementation.

## Discussion

This investigation provides comprehensive evidence that HT-LM1004 supplementation improves natural killer (NK) cell function in healthy adults, sustaining immune balance, with this enhancement closely associated with significant alterations in gut microbiota composition and metabolite profiles. Utilizing an integrated strategy that combined a randomized controlled trial with a spatially resolved *in vitro* human intestinal microbiome model, we established the immunostimulatory potential of HT-LM1004 and delineated a possible mechanistic link between probiotic-mediated microbial modulation and systemic immune responses.

In this randomized, double-masked, placebo-controlled clinical trial, we showed that 8 weeks of daily supplementation with HT-LM1004 significantly increased natural killer (NK) cell–mediated cytotoxic activity in healthy adults. This investigation demonstrates that HT-LM1004 supplementation significantly enhanced NK cell activity compared with the control group, as assessed by covariate-adjusted models. In both PP and ITT populations, ANCOVA-adjusted between-group differences were statistically significant across multiple effector-to-target (E:T) ratios (6.25:1, 12.5:1, 25:1, and 50:1), even after controlling for baseline variability and hyperlipidemia status (HLD). The results provide robust, quantitative clinical evidence supporting HT-LM1004’s ability to stimulate key components of the innate immune system, particularly NK cell function, which is crucial in the initial defense against viral infections and tumor development. Prior preclinical and limited clinical investigations have described the immunostimulatory properties of various *L. plantarum* strains, including HT-LM1004, reporting their capacity to boost NK cell activity and influence cytokine profiles [26, 27]. Interestingly, in the present study, slight improvements in certain immune parameters were also observed within the placebo group. Such responses are not uncommon in clinical trials involving healthy individuals, often attributed to the “placebo effect” or unintentional changes in lifestyle and dietary habits during the study period. Nevertheless, our between-group statistical analysis (ANCOVA), which adjusted for baseline values, demonstrated that the HT-LM1004 group achieved a significantly superior increase in NK cell activity and WBC counts compared to the placebo group. This confirms that the observed immunomodulatory effects are specifically driven by HT-LM1004 supplementation, beyond the minor physiological fluctuations observed in the control group. However, many past studies used single-ratio cytotoxicity assays or relied on surrogate immunological markers, which limited their ability to thoroughly characterize NK cell function. In contrast, our study utilized a multi-ratio, ex vivo cytotoxicity assay, providing a detailed, dose-dependent assessment of NK cell responses and enabling a more nuanced interpretation of immune activation. This methodological advancement allowed us to both verify and expand upon earlier reports by demonstrating a consistent elevation in NK cell cytotoxicity across a spectrum of immune stimulation levels. Employing several E:T ratios further enhanced the clinical significance of our results. In vivo, the NK-to-target ratio fluctuates and depends on tissue environment, infection status, or tumor burden. By demonstrating effectiveness across both low and high ratios, our data indicate that HT-LM1004 may increase NK cell responsiveness under diverse immunological scenarios. Furthermore, the observed magnitude of increase—from 12.4% to 19.2% depending on the ratio—represents a substantial improvement in cytotoxic capacity, potentially resulting in better initial protection against infections or cancerous changes. Interestingly, significant differences were observed in IL-12 and WBC as secondary outcomes by using ANCOVA with hyperlipidemia. However, a general downward trend in IL-12 in both placebo and treated groups is not unusual. Both groups had decreased IL-12 after treatment, but the experimental group’s reduction was larger and the between-group difference was statistically significant. Although IL-12 levels decreased in both the placebo and experimental groups over time, the reduction was significantly greater in the experimental group, suggesting a potential immunomodulatory effect of the intervention. The confidence intervals for some interleukins were wider than the corresponding point estimates, reflecting the inherent biological variability of cytokine responses. Importantly, cytokine outcomes were included as supportive mechanistic biomarkers rather than primary endpoints, and the main conclusions of the study are based on the observed changes in NK cell activity. It should be noted that the serum cytokine analysis in this study was conducted as an exploratory investigation to provide supportive mechanistic insights. Accordingly, no formal multiple testing correction was applied to the cytokine *p*-values to avoid the risk of missing potentially relevant biological trends (Type II error) in this healthy cohort. These findings are intended to complement the primary efficacy measure—NK cell activity—which remains the central focus and robust evidence of HT-LM1004's immunomodulatory potential. In this study, while HT-LM1004 supplementation significantly enhanced NK cell cytotoxic activity, no statistically significant changes were observed in the serum levels of cytokines such as IFN-γ and TNF-α. This discrepancy may be attributed to the complex and multi-faceted regulatory mechanisms of the innate immune system. NK cell activation can be triggered through various pathways, including direct cell-to-cell contact or modulation by other signaling molecules, which may not always result in a detectable increase in systemic cytokine concentrations within the peripheral blood. Furthermore, the timing of blood sampling relative to the transient peak of cytokine release might have influenced these observations. These findings suggest that HT-LM1004 may primarily enhance the effector function and lytic potential of NK cells rather than inducing a broad systemic inflammatory cytokine response, thereby supporting immune homeostasis while bolstering antiviral and antitumoral surveillance.

The absence of significant changes in immune stimulating-related cytokines indicates that HT-LM1004 may potentiate innate immunity while maintaining overall immune stability, aligning with earlier studies that reported decreased inflammatory markers following probiotic administration [28]. Mechanistically, the enhancement of NK cell activity by HT-LM1004 could arise from interactions between heat-inactivated structural components—such as peptidoglycan, lipoteichoic acid, and surface proteins—and pattern recognition receptors (PRRs), including Tolllike receptors (TLRs) on immune cells (Fig. S2). Such interactions are proposed to initiate downstream signaling involving mitogen-activated protein kinases (MAPKs), AP-1, and nuclear factor-kappa B (NF-κB), which promote the expression of cytotoxic mediators, notably perforin, granzyme B, and IFN-γ [26, 29]. Although our present investigation did not assess these pathways directly, the findings corroborate established mechanistic hypotheses, suggesting that future studies employing immunophenotyping and transcriptomic analyses could substantiate this mechanism. Clinically, selective activation of NK cells carries considerable translational relevance. NK cells function as pivotal agents in immune surveillance, particularly for early detection and elimination of virally infected or neoplastic cells. Their contributions are well documented in controlling infections with herpesviruses, influenza, hepatitis, and in restricting tumorigenesis at early stages [30]. Augmenting NK cell function through safe, non-pharmacological modalities—such as heat-inactivated probiotics—represents a potentially effective approach to bolster immune defense, especially in vulnerable groups such as older adults, individuals exposed to chronic stress, or those recuperating from viral diseases [31]. The favorable safety profile of HT-LM1004 documented in this trial further supports its potential as a functional ingredient suitable for extended use. No severe adverse events were detected, and laboratory assessments, vital signs, and participant-reported health status all revealed no clinically relevant abnormalities. Mild respiratory complaints occurred at similar rates between groups and were characteristic of common seasonal colds instead of being attributed to the intervention. Additionally, HT-LM1004 exhibited no negative impact on metabolic markers, including lipid profiles and liver enzyme levels, demonstrating its metabolic neutrality and supporting its applicability for a diverse population.

A methodological strength of this study is the implementation of a simulated human intestinal microbiome ecosystem, which provides an *in vitro* platform for investigating gut microbial dynamics. Conventional *in vitro* fermentation systems or animal models frequently lack adequate spatial and temporal resolution, thereby restricting their translational accuracy. In contrast, multi-compartment simulators are capable of replicating discrete colonic segments under controlled environmental conditions, supporting stable, long-term maintenance of donor-derived microbial communities [32-34]. This approach was crucial for placing the clinical observations of increased NK cell activity in an appropriate biological context. The randomized controlled trial revealed significant immunostimulatory effects without evidence of systemic inflammation, but the underlying biological mechanism remained to be elucidated. By recreating a region-specific, continuous intestinal environment, the simulator enabled investigation of the interactions between HT-LM1004 and complex microbial communities under physiologically relevant conditions[33, 35]. This finding supports the view that the immune benefits associated with HT-LM1004 result from a combination of direct activation of immune receptors by bacterial components [36] and indirect modulation via microbiome community restructuring [11, 33, 35]. Notably, the use of this model underscores a methodological progression in probiotic and postbiotic research, delivering a reproducible and ethically preferable platform for mechanistic investigation, as well as minimizing dependence on animal experimentation [11, 33, 35]. In the context of HT-LM1004, the model supports the interpretation that its immunostimulatory effects observed in humans are mediated through its ability to alter the intestinal microbial environment.

Another finding of this study is that HT-LM1004 supplementation produced compartment-specific changes in microbial diversity within the simulated human intestinal microbiome ecosystem. Alpha-diversity analyses revealed significant increases in species richness in the transverse and descending colon, while the ascending colon exhibited more consistent enhancements in diversity indices. These findings imply that HT-LM1004 induces a proximal-to-distal gradient effect, initially modifying species evenness in the proximal colon and subsequently promoting the expansion of low-abundance taxa in downstream segments. This pattern aligns with the ecological concept that microbial interventions frequently generate localized shifts that are transmitted along the intestinal tract via cross-feeding and metabolite exchange [11, 12]. Additional β-diversity and taxonomic analyses confirmed that HT-LM1004 supplementation led to clear separation of microbial communities from those in controls and promoted selective enrichment of rare taxa. Notably, some of these low-abundance genera have been suggested to be associated with ecosystem stability and metabolic functions despite their relatively low abundance [37, 38]. In the TC compartment, Clostridia were increased, supporting intestinal homeostasis through short-chain fatty acid production and enhanced epithelial barrier integrity [39]. The propionate-producing family Dialisteraceae was also enriched, consistent with its metabolic and immunomodulatory functions [40]. Additionally, Lentilactobacillus increased at the End and Post time points, a genus known for strengthening epithelial barriers and modulating host immunity [41]. In the DC compartment at the end time point, enrichment of low-abundance genera was likewise observed. Notably, members of Bacteroidales, Rikenellaceae, Lactonifactor, and Christensenella were associated with polysaccharide utilization, immune regulation, anti-inflammatory effects, microbial stability, and improved host metabolic health [42, 43]. The continued presence of these enriched taxa in the distal colon after HT-LM1004 was discontinued suggests that its ecological effects are enduring, possibly by expanding available niches or facilitating beneficial microbial networks. From a translational standpoint, these data support an ecological explanation for the immunological improvements noted in the clinical trial. Increased microbial diversity, particularly the rise of rare yet functionally critical taxa, is associated with greater immune resilience and a lower risk of dysbiosis-related disorders [1, 44]. In addition, β-diversity alterations suggest that HT-LM1004 reorganizes the overall microbial community structure rather than simply increasing dominant commensals. Such targeted and long-lasting restructuring may drive the microbiota–immune axis, explaining how HT-LM1004 augments NK cell activity without prompting systemic inflammation. While our previous clinical trial (IRB no. CUH 2020-12-012, Ref. 10) established the basic safety and preliminary immunomodulatory effects of HT-LM1004, the present study (IRB no. DAUHIRB-23-152) was designed to provide a more comprehensive understanding of its systemic impact. Specifically, unlike the previous study, we integrated a spatially resolved human intestinal microbiome simulator (SHIME^®^ system) to elucidate the ecosystem-level mechanisms. This approach allowed for the first time an in-depth investigation into compartment-specific changes in microbial diversity and metabolite profiles—such as secondary bile acids and short-chain fatty acids (SCFAs)—providing a mechanistic link between gut microbiome modulation and the observed enhancement of NK cell activity. In summary, HT-LM1004 supplementation not only enhanced microbial diversity in a compartment-dependent manner but also facilitated the proliferation of low-abundance, potentially beneficial taxa, thus altering the intestinal ecosystem in ways that likely contribute to its observed immunostimulatory effects in humans.

HT-LM1004 strain was cultured using a two-stage fermentation process. Initially, a seed culture was prepared in a medium containing glucose, soy peptone, and yeast extract at 34 ± 0.5°C for 6–8 h. This was followed by main fermentation in an 1,800 L bioreactor using an optimized medium (6.6% glucose, 1.3% soy peptone, 1.5% yeast extract, and mineral salts) at 34 ± 0.5°C for 15–18 h under nitrogen gas purging to maintain an anaerobic environment. After reaching a bacterial concentration of approximately 1.6 × 10^10^ CFU/mL, the biomass was harvested by centrifugation at 12,000 rpm. For the production of HT-LM1004, the recovered cells were mixed with cryoprotectants and subjected to heat treatment at 90°C for 30 min for complete inactivation. The heat-treated suspension was then freeze-dried and standardized. The final HT-LM1004 powder used in this study contained 2 × 10^11^ cells/g, ensuring a standardized dose of 2 × 10^10^ cells per 0.1 g capsule. Each active capsule contained 500 mg of HT-LM1004 powder, formulated under Good Manufacturing Practice (GMP) conditions. The daily dose consisted of 0.1 g of the standardized HT-LM1004 ingredient, which was blended with excipients including maltodextrin and magnesium stearate to reach the total capsule weight. The placebo capsules were identical in appearance and weight but contained only the excipients without the heat-treated postbiotic.

Another significant discovery from this study is that HT-LM1004 supplementation led to compartment-specific alterations in intestinal metabolite profiles, with bile acid metabolism becoming predominant in the proximal and transverse colon, and more extensive non–bile acid metabolic changes occurring in the distal colon. In the ascending colon, there was an increase in primary bile acids such as cholic acid and chenodeoxycholic acid, indicating augmented turnover of bile acids by microbial or host processes at the proximal entry site. Conversely, the transverse colon exhibited a marked enrichment of secondary bile acids, including deoxycholic acid and related derivatives, which signals active microbial transformation [45, 46]. Members of the *Lactiplantibacillus plantarum* species are known to possess bile salt hydro-lase (BSH) activity, which catalyzes the deconjugation of conjugated primary bile acids into their free forms [47]. These metabolic changes align with previous findings that primary bile acid activity predominates in proximal intestines, while secondary modifications occur in more distal regions driven by specific bacterial populations [48]. Notably, bile acids are now recognized as key immunomodulatory molecules that function via receptors such as FXR and TGR5 on both innate and adaptive immune cell populations [49, 50]. Specifically, secondary bile acids are involved in modulating natural killer T cell recruitment and their cytotoxic properties [51]. Therefore, the distinct bile acid profiles observed following HT-LM1004 supplementation suggest a potential mechanistic basis for the increased NK cell activity reported in the clinical phase of this work. In addition to bile acid metabolism, HT-LM1004 supplementation modified numerous non–bile acid metabolites, such as phenolic derivatives and small organic acids, especially in the distal colon. These metabolites are established modulators of epithelial barrier function, oxidative stress mitigation, and mucosal immune processes [52, 53]. This specific inclusion criterion was established to recruit individuals with a relatively compromised immune baseline or heightened susceptibility to physiological immune fluctuations. By targeting a population with a history of recurrent upper respiratory tract infections (URTIs), we aimed to more sensitively evaluate the clinical efficacy of HT-LM1004 in bolstering innate immune surveillance and resilience. This strategic selection ensures that the study assesses the immunomodulatory potential of the postbiotic in a cohort that stands to derive the most significant clinical benefit from such supplementation. The accumulation of gingerol and coumarate derivatives, for example, suggests that HT-LM1004-mediated cross-feeding may broaden the functional metabolic capacity of the microbiome. Collectively, these results indicate that HT-LM1004 does not induce uniform metabolic changes, but rather drives region-specific reorganization of bile acid and non–bile acid pathways, which could collectively promote enhanced host immune surveillance.

This study further demonstrated that HT-LM1004 supplementation led to dynamic shifts in short-chain fatty acid (SCFA) production, dependent on both gut compartment and time. In the ascending colon, SCFA concentrations temporarily declined during the mid-intervention phase but returned to baseline or exceeded initial levels in the late and post-supplementation stages. Conversely, the transverse and descending colon consistently showed elevated levels of acetate, propionate, butyrate, and valerate during supplementation, with only partial reductions after HT-LM1004 withdrawal. These temporal and spatial distinctions underscore the intricate ecological responses of the gut microbiota to postbiotic exposure, in which transient suppression may indicate microbial adaptation. At the same time, subsequent enrichment is likely driven by cross-feeding interactions and the proliferation of fermentative microbiota [54, 55]. The increases in butyrate and propionate have notable significance, given that these metabolites possess potent immunoregulatory effects. Butyrate facilitates the cytotoxic response of NK and CD8+ T cells through epigenetic mechanisms and stimulates regulatory T cell differentiation [56, 57], whereas propionate is associated with the regulation of inflammatory signaling and preservation of the mucosal barrier [58]. Acetate, which is the predominant SCFA, is involved in systemic metabolic modulation and supports antiviral immunity [59]. As a result, the compartment-specific SCFA enrichment identified in this investigation provides a credible mechanism for the selective increase in NK cell activity reported in the clinical study, without provoking systemic inflammatory responses. Collectively, these results reinforce the hypothesis that HT-LM1004 supplementation drives a targeted transformation of microbial metabolic outputs, resulting in temporally and regionally distinct SCFA alterations. Such changes are likely integral to the microbiota–immune axis that facilitates enhanced host immune defense associated with HT-LM1004. Thus, findings from the microbiome-only system provide mechanistic and ecological insights into how HT-LM1004 may influence the gut microbial environment and SCFA production that are likely associated with the enhanced NK cell activity.

The integrative analysis of our clinical and *in vitro* findings indicates that the immunostimulatory effects of HT-LM1004 operate via a dual mechanism engaging both direct and indirect pathways. HT-LM1004 preserves structural elements, including peptidoglycan and lipoteichoic acids, which directly interact with pattern recognition receptors such as TLR2 on immune cells. This engagement initiates the activation of MAPK and NF-κB signaling pathways, subsequently elevating cytotoxic molecules such as perforin and granzyme B [8]. Also, the activation of cytotoxic mediators such as perforin and granzyme B by HT-LM1004 was investigated (Fig. S1) and this direct mechanism had been suggested through iNK cells imbedded in intestinal epithelial cells [30]. Another direct mechanism have been demonstrated in other heat-killed or postbiotic *L. plantarum* strains, which have been shown to improve NK cell cytotoxicity and regulate cytokine secretion *in vitro* and *in vivo* [60]. Concurrently, results from the simulated intestinal ecosystem indicate that HT-LM1004 administration alters the gut microbiome, selectively increasing low-abundance taxa and inducing metabolic changes characterized by elevated secondary bile acids and SCFAs. These metabolites function as immunomodulators: secondary bile acids influence NK and NKT cell migration and activity through FXR and TGR5 signaling [3, 46, 61], whereas butyrate and propionate facilitate NK and CD8^+^ T cell cytotoxicity by inhibiting histone deacetylase and supplying metabolic substrates [54, 57, 59]. Comparable microbiota–immune interactions have been documented in studies where increased SCFA production following dietary fiber or probiotic supplementation augmented antiviral immunity or enhanced tumor immunosurveillance through NK cell activation [62]. Collectively, HT-LM1004 appears to enhance NK cell activity through synergistic mechanisms involving postbiotic structural signaling and microbiota-derived metabolite modulation, supporting a mechanistic basis for the observed clinical potentiation of immune response without provoking systemic inflammation.

This study presents several limitations. The clinical trial included only healthy adults, restricting the generalizability of results to vulnerable groups such as the elderly or immunocompromised individuals. Although the SHIME model offers insights into underlying mechanisms, it does not fully replicate host–microbe and immune system interactions *in vivo*. Additionally, associations observed among microbiome remodeling, metabolite changes, and NK cell activation are correlational rather than demonstrably causal. The relatively brief intervention period further limits assessment of the longevity of these effects. Future research involving more diverse cohorts, extended follow-up durations, and direct causal validation will be necessary. In conclusion, despite these constraints, our findings provide strong clinical and mechanistic evidence that HT-LM1004 enhances NK cell activity via both direct immune receptor interaction and microbiome–metabolite modulation. By integrating data from a randomized trial with ecosystem-level models, this study establishes a translational framework for evaluating postbiotics as advanced immunomodulatory agents. The clinical and mechanistic evidence presented here underscores the translational potential of HT-LM1004 in the burgeoning postbiotics industry. As a heat-inactivated preparation, HT-LM1004 offers significant industrial advantages, including superior shelf-stability and safety for immunocompromised individuals compared to live probiotics. These characteristics make it an ideal candidate for diverse functional food formats—such as beverages, powders, or capsules—specifically formulated to bolster NK cell-mediated immune surveillance in populations susceptible to immune senescence or stress-induced immunosuppression. Moreover, our spatially-resolved microbiome data suggest that HT-LM1004 could serve as a core component of precision microbiome-based therapeutics aimed at restoring intestinal homeostasis through targeted metabolite modulation, providing a safe and effective option for clinical supportive care without the risks associated with viable microbial translocation.

Though there is no direct mechanistic linkage between microbiome and metabolomics datasets, the observed enrichment of specific microbial taxa, together with alterations in SCFA- and bile acid-associated metabolic pathways, suggests a potential association with immune enhancement. While further research including comparison between changes in SCFA concentrations and microbiome composition observed in the simulator system and those detected in human stool samples, is necessary to validate the physiological relevance of the simulator-based findings. Additional comprehensive studies are also required to fully elucidate the direct causal relationships between these *in vitro* microbial alterations and systemic immune responses. Our findings provide complementary evidence from both clinical and ecosystem-level models, supporting the role of HT-LM1004 as a multi-target immunomodulator.

## Conclusion

**Fig. 7:**
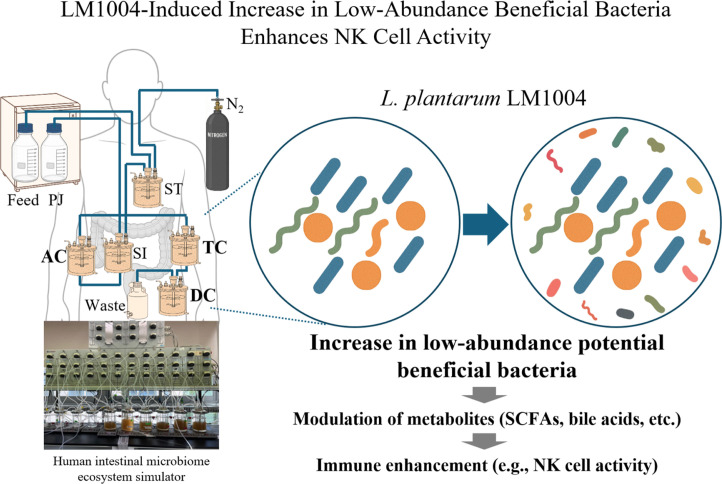
Graphical summary of the effects of HT-LM1004 on the gut microbiota. The schematic representation of the Simulator of the Human Intestinal Microbial Ecosystem depicts the influence of HT-LM1004 supplementation on gut microbial community structure. HT-LM1004 administration increased the relative abundance of minor potential beneficial bacteria in the colon, particularly at the endpoint (4 weeks of supplementation) and the postpoint (1.5-week recovery), with pronounced effects in the transverse colon (TC) and descending colon (DC). These microbiota shifts coincided with alterations in metabolite production, such as short-chain fatty acids (SCFAs) and bile acids, which are believed to contribute to the enhancement of natural killer (NK) cell activity identified in the clinical trial.

This study establishes that HT-LM1004 is both efficacious and safe in augmenting the immune response, as demonstrated by a significant enhancement of NK cell activity that sustains immune balance in a randomized, placebo-controlled trial. Beyond clinical validation, mechanistic investigations using a simulated human intestinal microbiome ecosystem revealed that HT-LM1004 preferentially enriched low-abundance taxa that have been previously linked to potential health-related functions, improved microbial diversity throughout colon regions, and modulated compartment-specific metabolic pathways, particularly those involving bile acids and SCFAs. As depicted in the graphical representation (Fig. 7), these combined results underscore a dual mechanism by which HT-LM1004 restructures gut microbial communities and modifies their metabolic activities to facilitate immune enhancement. Notably, this comprehensive methodology, connecting human clinical outcomes with mechanistic elucidation, positions HT-LM1004 as a promising candidate for development as a next-generation microbiome-based preventative or functional food. Subsequent studies in older or immunocompromised subjects, and those incorporating prolonged intervention periods, will be essential to substantiate its translational relevance and advance the clinical application of microbiome-mediated immunomodulation.

## Supplemental Materials

Supplementary data for this paper are available on-line only at http://jmb.or.kr.



## Figures and Tables

**Table 1:** **Results of clinical trial.** (**A**) PP Analysis to Evaluate the After-Before Within-Individual Change in Each Group (**B**) PP Analysis of Covariance with Covariates to Evaluate the Treatment-Control Between-Group Difference (**C**) ITT Analysis to Evaluate After-minus-Before Within-Individual Changes in Each Group (**D**) ITT Analysis of Covariance with the Hyperlipidemia Status as the Covariate to Evaluate the Treatment-Control Between-Group Difference.