# Supplementary material for: Microbiome-Modulating Effects of Heat-Treated Lactiplantibacillus plantarum LM1004 and Its Enhancement of NK Cell Activity: Evidence from a Clinical Trial and a Simulated Human Intestinal Microbiome Ecosystem
Source: J Microbiol Biotechnol. 2026 Jul 3;36:e2604046. doi: 10.4014/jmb.2604.04046 (PMC13396743; doi:10.4014/jmb.2604.04046)
Supplement: Supplementary file 1 [file jmb-36-e2604046-supple.pdf]

J. Microbiol. Biotechnol. 2026. 36: e2604046

https://doi.org/10.4014/jmb.2604.04046

Supplementary Tables and Figures

Table S1. The questionnaire used in this study for PSS measurement.

대상자 이니셜	대상자 스크리닝번호(SN)	방문일자
	S- ________________	202_년 ___월 ___일

한국판 스트레스 자각척도(PSS)

다음의 문항들은 최근 1개월 동안 당신이 느끼고 생각한 것에 대한 것입니다.
각 문항에 해당하는 내용을 얼마나 자주 느꼈는지 표기해 주십시오.

1. 최근 1개월 동안, 예상치 못했던 일 때문에 당황했던 적이 얼마나 있었습니까?
⓪ 전혀 없었다. ① 거의 없었다. ② 때때로 있었다. ③ 자주 있었다. ④ 매우 자주 있었다.

2. 최근 1개월 동안, 인생에서 중요한 일들을 조절할 수 없다는 느낌을 얼마나 경험하였습니까?
⓪ 전혀 없었다. ① 거의 없었다. ② 때때로 있었다. ③ 자주 있었다. ④ 매우 자주 있었다.

3. 최근 1개월 동안, 신경이 예민해지고 스트레스를 받고 있다는 느낌을 얼마나 경험하였습니까?
⓪ 전혀 없었다. ① 거의 없었다. ② 때때로 있었다. ③ 자주 있었다. ④ 매우 자주 있었다.

4. 최근 1개월 동안, 당신의 개인적 문제들을 다루는 데 있어서 얼마나 자주 자신감을 느꼈습니까?
⓪ 전혀 없었다. ① 거의 없었다. ② 때때로 있었다. ③ 자주 있었다. ④ 매우 자주 있었다.

5. 최근 1개월 동안, 일상의 일들이 당신의 생각대로 진행되고 있다는 느낌을 얼마나 경험하였습니까?
⓪ 전혀 없었다. ① 거의 없었다. ② 때때로 있었다. ③ 자주 있었다. ④ 매우 자주 있었다.

6. 최근 1개월 동안, 당신이 꼭 해야 하는 일을 처리할 수 없다고 생각한 적이 얼마나 있었습니까?
⓪ 전혀 없었다. ① 거의 없었다. ② 때때로 있었다. ③ 자주 있었다. ④ 매우 자주 있었다.

7. 최근 1개월 동안, 일상생활의 짜증을 얼마나 자주 잘 다스릴 수 있었습니까?
⓪ 전혀 없었다. ① 거의 없었다. ② 때때로 있었다. ③ 자주 있었다. ④ 매우 자주 있었다.

8. 최근 1개월 동안, 최상의 컨디션이라고 얼마나 자주 느끼셨습니까?
⓪ 전혀 없었다. ① 거의 없었다. ② 때때로 있었다. ③ 자주 있었다. ④ 매우 자주 있었다.

9. 최근 1개월 동안, 당신이 통제할 수 없는 일 때문에 화가 난 경험이 얼마나 있었습니까?
⓪ 전혀 없었다. ① 거의 없었다. ② 때때로 있었다. ③ 자주 있었다. ④ 매우 자주 있었다.

10. 최근 1개월 동안, 어려운 일들이 너무 많이 쌓여서 극복하지 못할 것 같은 느낌을 얼마나 자주 경험하셨습니까? ⓪ 전혀 없었다. ① 거의 없었다. ② 때때로 있었다. ③ 자주 있었다. ④ 매우 자주 있었다.

총점	

작성자 확인	202_ 년 월 일 (서명)

Factor loadings for analysis

	Abbreviated item description	*KPPS-14* Factor 1 NS	*KPPS-14* Factor 2 PS	*KPPS-10* Factor 1 NS	*KPPS-10* Factor 2 PS	*KPPS-4* Factor 1 PS	*KPPS-4* Factor 2 NS
1	Upset because of something that happened unexpectedly	**0.79**	–0.06	**0.79**	–0.03		
2	Unable to control the important things in your life	**0.76**	0.05	**0.78**	0.05	0.06	**0.68**
3	Nervous or stressed	**0.77**	–0.11	**0.78**	–0.08		
8	Not coping with all the things you have to do	**0.47**	–0.11	**0.48**	–0.14		
11	Anger because of things that happened that are outside of your control	**0.75**	–0.01	**0.75**	0.01		
12	Thinking about things that you have to accomplish	**0.74**	–0.20				
14	Difficulties are piling up so high that you cannot overcome them	**0.73**	–0.06	**0.7**	–0.01	0.02	**0.76**
4	Dealing successfully with day-to-day problems and annoyances	–0.12	0.75				
5	Effectively coping with important changes that are occurring in your life	–0.14	0.7				
6	Confident about your ability to handle your personal problems	0.10	**0.75**	0.08	**0.76**	**0.71**	0.08
7	Things are going your way	0.05	**0.68**	0.02	**0.65**	**0.74**	0.01
9	Able to control irritations in your life	–0.19	**0.54**	–0.22	**0.52**		
10	You are on top of things	–0.04	**0.72**	–0.05	**0.80**		
13	Able to control the way you spend your time	–0.15	**0.63**				
	Eigenvalue	4.24	2.88	3.2	1.91	1.06	1.05
	Percentage of variance explained	30.28	20.58	32.04	19.07	26.42	26.19

KPPS-14, Korean version of the Perceived Stress Scale-14; KPPS-10, Korean version of the Perceived Stress Scale-10; KPPS-4, Korean version of the Perceived Stress Scale-4; NS, negative subscale; PS, positive subscale. Boldface values represent significant loadings.

The PSS (Perceived Stress Scale) was used exclusively as a screening tool to confirm eligibility at enrollment; Participants were enrolled only if they met the predefined inclusion criterion (PSS ⩾ 16, corresponding to moderate stress according to Korean standards).

Table S2. Compliance rates in the ITT and the PP populations.

	ITT population			PP population		
Variable	Control (n=60)	Experimental (n=59)	*p*-value**	Control (n=55)	Experimental (n=57)	*p*-value**
Visit 2–3	97.75 ± 4.45	97.74 ± 4.16	0.479	98.25 ± 3.65	97.66 ± 4.21	0.212
Visit 3–4	99.04 ± 2.39	98.32 ± 3.97	0.487	99.04 ± 2.39	98.29 ± 4.00	0.460
Visit 2–4	98.60 ± 2.70	97.93 ± 3.48	0.157	98.60 ± 2.70	97.90 ± 3.50	0.138

Data are presented as mean ± standard deviation. Normality was assessed using the Shapiro–Wilk test. ***P* values indicate comparisons between groups: Mann–Whitney *U* test.

Table S3. Baseline characteristics of study participants in in the ITT and the PP populations.

		ITT population			PP population		
Variable		Control (n=60)	Experimental (n=59)	*p*-value	Control (n=55)	Experimental (n=57)	*p*-value
Age (years)		56.17 ± 11.80	57.07 ± 10.50	0.708²	56.40 ± 12.01	57.00 ± 10.62	0.845²
Height (cm)		158.53 ± 6.36	159.40 ± 7.36	0.617²	158.68 ± 6.51	159.10 ± 7.16	0.841²
Weight (kg)		58.94 ± 8.43	60.17 ± 8.83	0.706²	58.79 ± 8.43	59.84 ± 8.77	0.839²
Body temperature (°C)		36.52 ± 0.13	36.48 ± 0.08	0.101²	36.53 ± 0.13	36.48 ± 0.09	0.048² *
Diastolic BP (mmHg)		75.62 ± 9.30	75.14 ± 9.90	0.785¹	74.87 ± 9.00	75.28 ± 10.01	0.821¹
Systolic BP (mmHg)		128.08 ± 14.41	127.97 ± 14.13	0.964¹	127.44 ± 14.35	128.02 ± 14.34	0.831¹
Pulse rate		75.07 ± 8.47	75.81 ± 8.77	0.637¹	75.15 ± 8.62	76.18 ± 8.64	0.529¹
Sex	Male	7 (11.7%)	9 (15.3%)	0.566³	7 (12.7%)	8 (14.0%)	0.839³
	Female	53 (88.3%)	50 (84.7%)		48 (87.3%)	49 (86.0%)	
Smoking status	Non-smoker	57 (95.0%)	51 (86.4%)	0.129⁴	52 (94.5%)	50 (87.7%)	0.492⁴
	Former smoker	2 (3.3%)	7 (11.9%)		2 (3.6%)	6 (10.5%)	
	Current smoker	1 (1.7%)	1 (1.7%)		1 (1.8%)	1 (1.8%)	
Alcohol consumption	Non-drinker	50 (83.3%)	49 (83.1%)	0.967³	46 (83.6%)	48 (84.2%)	0.934³
	Moderate drinker	10 (16.7%)	10 (16.9%)		9 (16.4%)	9 (15.8%)	
	Heavy drinker	0 (0.0%)	0 (0.0%)		0 (0.0%)	0 (0.0%)	

P values were calculated using 1) independent *t*-test, 2) Mann–Whitney U test, 3) Chi-square test, or 4) Fisher's exact test; * $p < 0.05$.

Table S4. Comparison of medical histories between groups in the ITT and the PP populations.

		ITT population			PP population		
Variable		Control (n=60)	Experimental (n=59)	*p*-value **	Control (n=55)	Experimental (n=57)	*p*-value**
Skin disorder	Yes	3(5.0%)	0(0.0%)	0.244[2)]	3(5.0%)	0(0.0%)	0.115[2)]
	No	57(95.0%)	59(100.0%)		52(95.0%)	57(100.0%)	
Musculoskeletal disorder	Yes	3(5.0%)	4(6.8%)	0.717[2)]	3(5.5%)	4(7.0%)	1.000[2)]
	No	57(95.0%)	55(93.2%)		52(94.5%)	53(93.0%)	
Ophthalmic disorder	Yes	1(1.7%)	0(0.0%)	1.000[2)]	1(1.8%)	0(0.0%)	0.491[2)]
	No	59(98.3%)	59(100.0%)		54(98.2%)	57(100.0%)	
Otic disorder	Yes	1(1.7%)	1(1.7%)	1.000[2)]	1(1.8%)	1(1.8%)	1.000[2)]
	No	59(98.3%)	58(98.3%)		54(98.2%)	56(98.2%)	
Sensory impairment	Yes	0(0.0%)	0(0.0%)	-	0(0.0%)	0(0.0%)	-
	No	60(100.0%)	59(100.0%)		55(100.0%)	57(100.0%)	
Neurological/Psychiatric disorder	Yes	0(0.0%)	3(5.0%)	0.119[2)]	0(0.0%)	3(5.3%)	0.243[2)]
	No	60(100.0%)	567(94.9%)		55(100.0%)	54(94.7%)	
Gastrointestinal disorder	Yes	0(0.0%)	0(0.0%)	-	0(0.0%)	0(0.0%)	-
	No	60(100.0%)	59(100.0%)		55(100.0%)	57(100.0%)	
Hepatic/Biliary disease	Yes	0(0.0%)	0(0.0%)	-	0(0.0%)	0(0.0%)	-
	No	60(100.0%)	59(100.0%)		55(100.0%)	57(100.0%)	
Metabolic/Nutritional disorder	Yes	0(0.0%)	0(0.0%)	-	0(0.0%)	0(0.0%)	-
	No	60(100.0%)	59(100.0%)		55(100.0%)	57(100.0%)	
Endocrine disease	Yes	5(8.3%)	11(18.6%)	0.099	5(9.1%)	10(17.5%)	0.189[1)]
	No	55(91.7%)	48(81.4%)		50(90.9%)	47(82.5%)	
Cardiovascular disorder	Yes	7(11.7%)	5(8.5%)	0.563[2)]	7(12.7%)	5(8.8%)	0.499[1)]
	No	53(88.3%)	54(91.5%)		48(87.3%)	52(91.2%)	
Immune disorder	Yes	0(0.0%)	0(0.0%)	-	0(0.0%)	0(0.0%)	-
	No	60(100.0%)	59(100.0%)		55(100.0%)	57(100.0%)	
Respiratory disorder	Yes	0(0.0%)	0(0.0%)	-	0(0.0%)	0(0.0%)	-
	No	60(100.0%)	59(100.0%)		55(100.0%)	57(100.0%)	
Hematologic disorder	Yes	0(0.0%)	0(0.0%)	-	0(0.0%)	0(0.0%)	-
	No	60(100.0%)	59(100.0%)		55(100.0%)	55(100.0%)	
Infectious disorder	Yes	0(0.0%)	0(0.0%)	-	0(0.0%)	0(0.0%)	-
	No	60(100.0%)	59(100.0%)		55(100.0%)	55(100.0%)	
Renal/Urinary disease	Yes	0(0.0%)	0(0.0%)	-	0(0.0%)	0(0.0%)	-
	No	60(100.0%)	59(100.0%)		55(100.0%)	57(100.0%)	
Surgical History	Yes	0(0.0%)	0(0.0%)	-	0(0.0%)	0(0.0%)	-
	No	60(100.0%)	59(100.0%)		55(100.0%)	57(100.0%)	
Hypertension	Yes	7(11.7%)	5(8.5%)	0.563[2)]	7(12.7%)	5(8.8%)	0.499[1)]
	No	53(88.3%)	54(91.5%)		48(87.3%)	52(91.2%)	
Hyperlipidemia	Yes	4(6.7%)	11(18.6%)	**0.049**[2)]	4(7.3%)	10(17.5%)	0.100[1)]
	No	56(93.3%)	48(81.4%)		51(92.7%)	47(82.5%)	
Osteoporosis	Yes	2(3.3%)	2(3.4%)	1.0000[2)]	2(3.6%)	2(3.5%)	1.0000[2)]
	No	58(96.7%)	57(96.6%)		53(96.4%)	55(96.5%)	
		53(96.4%)	55(96.5%)				

Data were expressed as frequency (proportion). 1) P values were derived from the chi-square test. 2) P values were derived from Fisher's exact test.

Table S5. Comparison of concomitant medication usage between groups in the ITT and the PP populations.

Variable		ITT population			PP population		
		Control (n=60)	Experimental (n=59)	*p*-value **	Control (n=55)	Experimental (n=57)	*p*-value**
Concomitant medication	No	46(76.7%)	46(78.0%)	0.866[1)]	41(74.5%)	45(78.9%)	0.581[1)]
	Yes	14(23.3%)	13(22.0%)		14(25.5%)	12(21.1%)	

Data were expressed as frequency (proportion). 1) P values were derived from the chi-square test.

Table S6. Baseline Concomitant Medication Status by Group.

Control Group (n=55)		
Subject ID	Medication	Indication
R020	Imotem Cap 300 mg	Rheumatoid arthritis
	Dicabon 500 Tab	
R022	Aximar Tab	Common cold
	Otillen Tab	
	Clopizin Tab	
R033	Tylenol 500 mg	Common cold
R034	Vimovo 500 mg	Rheumatoid arthritis
	Shinbaro 300 mg	
	Epesine SR 75 mg	
R036	Dicamax D Tab	Osteoporosis
R039	Olmetec Plus 20/12.5 mg	Hypertension
R047	LaCor 60/12.5 mg	Hypertension
	Livalozet 2/10 mg	Hyperlipidemia
	Banexin 80 mg	Prevention of hypertensive complications
	Fluconazole capsule	Tinea pedis
R052	Zeroid Intensive Lotion MD 300 mL	Xerosis
	Loteprednol ophthalmic Suspension 0.5% 4 mL	Bilateral keratitis
R067	Tramol Tab 325 mg	Common cold
	Mucoserine capsule	
	Motilitone Tab	
R071	Lockpain Tab	Common cold
	Arlicough Tab	
	Caster Tab	
	Peniramin 2 mg	
	Cough syrup 20 mL	
	Motilitone Tab	
R072	Cresnon Tab 5 mg	Hyperlipidemia
R074	Esuba Tab 10/5 mg	Hyperlipidemia
	Entelon Tab 150 mg	Congestive heart failure
	Choliatin Soft Cap	Cerebrovascular prevention
R104	Livaro 2 mg	Hyperlipidemia
	Losartan 50 mg	Hypertension
R107	Olmetec Tab 10 mg	Hypertension
R108	Varban Tab 160 mg	Hypertension

Experimental Group (n = 57)		
Subject ID	Medication	Indication
R014	Dicaduo Tab 100/1000	Osteoporosis
R015	Cresba Tab 10 mg	Hyperlipidemia
	Gria Soft Cap	Cognitive improvement
	Anfrade SR Tab 300 mg	Atherosclerosis prevention
R017	Norvasc Tab 5 mg	Hypertension
	Lipiwon Tab 10 mg	Hyperlipidemia
	Astrix 100 mg	Prevention of hypertensive complications
R021	Loxfen Tab	Common cold
	Sterin 60 mg	
	Mucidin Cap 200 mg	
R032	Forxiga Tab 10 mg	Diabetes
	Euglex Tab 2 mg	
	Diabex 500 mg	
	Simvastatin Tab. Dongsung	Hyperlipidemia
R037	Dichlozid Tab	Hypertension
	Cozaar Plus Tab 40 mg	
R057	Cozaar Plus	Hypertension
	Crestor Tab	Hyperlipidemia
R061	Pletal SR Cap 75 mg	Stroke
	Lipilou Tab 20 mg	Hyperlipidemia
R075	Pretabac capsule 100 mg	Cerebrovascular disease
	Rosuvamibe 10/10 mg	Hyperlipidemia
R078	Astrix Cap 100 mg	Prevention of hypertensive complications
	Stilnox Tab 10 mg	Insomnia prevention
	Sevikar Tab 5/20 mg	Hypertension
	Dicamax D Tab	Osteoporosis prevention
	Cycin Tab 250 mg	Bronchitis
R080	Davi Duo Tab 10/5 mg	Hyperlipidemia
	Anpl-one SR Tab 300 mg	Atherosclerosis prevention
R085	Simvastatin Tab 20 mg	Hyperlipidemia
	Cal-D Tec Chewable Tab.	Osteoporosis
R095	Twynsta 40/5 mg	Hypertension
	Lipilou Tab 20 mg	Hyperlipidemia
R106	Mevalotin 20 mg	Hyperlipidemia

		Platless Tab	Stroke
		Sermion Tab 10 mg	Cognitive improvement
	R110	Norvasc Tab 5 mg	Hypertension
	R115	Tylenol 500 mg	Common cold
	R120	Amosartan 5/50 mg	Hypertension
		Rosuzet Tab 10/10 mg	Hyperlipidemia
		Plavix Tab 75 mg	Prevention of hypertensive complications

Table S7. Detailed adverse events categorized by severity.

System Organ Class	Test group (N=59)						Control group (N=60)					
	Mild		Moderate		Severe		Mild		Moderate		Severe	
Preferred Term	n(%)	[F]	n(%)	[F]	n(%)	[F]	n(%)	[F]	n(%)	[F]	n(%)	[F]
Respiratory conditions	5(8.47)	[6]	0(0.00)	[0]	0(0.00)	[0]	4(6.67)	[4]	2(3.33)	[2]	0(0.00)	[0]
Common cold	5(8.47)	[6]	0(0.00)	[0]	0(0.00)	[0]	4(6.67)	[4]	1(1.67)	[1]	0(0.00)	[0]
Bronchiolitis episodes	0(0.00)	[0]	0(0.00)	[0]	0(0.00)	[0]	0(0.00)	[0]	1(1.67)	[1]	0(0.00)	[0]
Total cases	5(8.47)	[6]	0(0.00)	[0]	0(0.00)	[0]	4(6.67)	[4]	2(3.33)	[2]	0(0.00)	[0]

* [F] = frequency; Medical coding was performed according to WHO-ART (World Health Organization Adverse Reaction Terminology) version 092.

Table S8. Comparison of hematological and biochemical parameters within and between groups in the ITT population.

Parameter		Measured value			Difference from baseline				
		Control group (n=60)	Experimental group (n=59)	*p*-value **	Control arm	*p*-value*	Experimental group	*p*-value*	*p*-value**
**RBC (3.6~4.9x10^3/μL)**	Visit 1	4.26±0.32	4.35±0.35	0.179[2)]					
	Visit 2	4.35±0.35	4.40±0.35	0.419[1)]	0.09±0.19	**<0.001[3)]**	0.05±0.18	**0.014[4)]**	0.168[2)]
	Visit 3	4.33±0.32	4.40±0.38	0.264[2)]	0.07±0.19	**0.001[4)]**	0.04±0.23	0.075[4)]	0.425[2)]
**Hemoglobin (11~15g/dL)**	Visit 1	12.99±1.05	13.33±1.14	0.248[2)]					
	Visit 3	13.29±1.12	13.49±1.31	0.550[2)]	0.29±0.56	**<0.001[3)]**	0.17±0.53	**0.003[4)]**	0.256[2)]
	Visit 4	13.21±1.02	13.44±1.39	0.433[2)]	0.21±0.59	**0.007[3)]**	0.11±0.67	0.125[4)]	0.587[2)]
**HCT (33~44%)**	Visit 1	38.21±2.95	39.12±2.97	0.241[2)]					
	Visit 3	39.18±3.11	39.73±3.15	0.389[2)]	0.97±1.66	**<0.001[3)]**	0.62±1.74	**0.001[4)]**	0.323[2)]
	Visit 4	38.87±2.69	38.60±3.44	0.278[2)]	0.66±1.67	**0.003[3)]**	0.49±2.04	**0.035[4)]**	0.605[1)]
**Platelet (14.0~10^3/uL)**	Visit 1	246.15±59.33	242.63±48.41	0.907[2)]					
	Visit 3	247.88±57.02	247.80±48.51	0.754[2)]	1.73±21.73	0.462[4)]	5.17±19.58	0.065[4)]	0.579[2)]
	Visit 4	253.02±57.89	247.47±49.15	0.898[2)]	6.87±29.53	0.051[4)]	4.85±24.37	0.132[4)]	0.657[2)]
**Neutrophil (40~70%)**	Visit 1	54.01±9.36	54.42±8.52	0.803[1)]					
	Visit 3	50.58±9.65	53.90±9.60	0.062[1)]	-3.43±9.21	**0.005[3)]**	-0.52±6.42	0.606[3)]	0.288[2)]
	Visit 4	54.81±8.06	54.41±10.10	0.339[1)]	0.80±7.63	0.422[3)]	1.99±8.47	0.076[3)]	0.543[2)]
**Lymphocyte (20~47%)**	Visit 1	36.57±8.42	35.98±8.07	0.696[1)]					
	Visit 3	39.04±9.42	35.95±8.48	0.062[1)]	2.47±8.40	**0.026[3)]**	-0.03±6.42	0.971[3)]	0.199[2)]
	Visit 4	35.53±7.69	34.28±9.17	0.424[1)]	-1.05±6.84	0.241[3)]	-1.70±7.13	0.073[3)]	0.790[2)]
**Monocyte (3~10%)**	Visit 1	6.64±1.49	6.69±1.27	0.844[1)]					
	Visit 3	7.31±1.57	7.21±1.41	0.739[1)]	0.66±1.14	**0.001[3)]**	0.52±1.08	**<0.001[3)]**	0.489[1)]
	Visit 4	6.77±1.64	6.75±1.62	0.869[2)]	0.13±1.27	0.504[4)]	0.05±1.32	0.753[3)]	0.761[1)]
**Eosinophil (0~7%)**	Visit 1	2.17±1.26	2.21±1.60	0.772[2)]					
	Visit 3	2.34±1.27	2.22±1.81	0.186[2)]	0.17±0.88	0.179[4)]	0.01±1.31	0.975[3)]	0.473[2)]
	Visit 4	2.27±1.45	1.99±1.54	0.227[2)]	0.10±1.13	0.880[4)]	0.22±1.11	0.233[3)]	0.482[2)]
**Basophil (0~2%)**	Visit 1	0.61±0.37	0.70±0.39	0.205[2)]					
	Visit 3	0.73±0.33	0.72±0.37	0.460[2)]	0.13±0.29	**0.001[3)]**	0.02±0.27	0.679[4)]	0.052[2)]
	Visit 4	0.63±0.37	0.57±0.37	0.318[2)]	0.02±0.34	0.706[4)]	-0.13±0.41	**0.028[4)]**	0.058[2)]
**ESR (0~30mm/hr)**	Visit 1	9.75±7.02	12.41±9.90	0.258[2)]					
	Visit 3	8.57±7.25	10.25±8.13	0.188[2)]	-1.18±5.52	**0.038[4)]**	-2.15±6.86	**0.017[4)]**	0.841[2)]
	Visit 4	11.50±8.09	12.46±8.77	0.526[2)]	1.75±6.92	**0.046[4)]**	0.05±6.58	0.450[4)]	0.380[2)]
**Glucose (74~106mg/dL)**	Visit 1	96.07±8.80	99.81±12.22	0.087[2)]					
	Visit 3	96.73±14.29	99.31±14.41	0.958[2)]	0.67±13.31	**0.028[4)]**	-0.51±6.89	**0.172[4)]**	**0.016[2)]**
	Visit 4	96.97±7.83	13.44±1.39	0.486[2)]	0.90±6.85	0.313[3)]	0.27±8.23	0.827[4)]	0.491[2)]
**BUN (7~20mg/dL)**	Visit 1	14.23±4.27	14.19±3.96	0.877[2)]					
	Visit 3	13.95±3.99	13.90±3.30	0.885[2)]	-0.28±3..14	0.611[4)]	-0.29±3.47	0.484[4)]	0.994[1)]
	Visit 4	14.28±3.97	14.41±3.53	0.906[2)]	0.05±3.35	0.884[4)]	0.22±4.03	0.693[4)]	0.802[1)]
**Creatinine (0.51~0.95mg/dL)**	Visit 1	0.70±0.10	0.75±.0.14	0.052[2)]					
	Visit 3	0.71±0.11	0.75±0.14	0.077[2)]	0.01±0.07	0.382[4)]	0.00±0.06	0.464[4)]	0.257[1)]
	Visit 4	0.69±0.11	0.75±0.15	0.058[2)]	-0.01±0.06	0.448[4)]	0.00±0.07	0.465[4)]	0.983[1)]
**Total cholesterol (0 ~200mg/dL)**	Visit 1	189.17±4.46	203.32±46.21	0.059[1)]					
	Visit 3	193..22±34.24	206.56±47.61	0.083[1)]	4.15±17.59	0.073[3)]	3.24±19.52	0.208[3)]	0.789[1)]
	Visit 4	193.95±32.73	205.47±44.02	0.108[1)]	4.88±25.84	0.149[3)]	2.15±27.52	0.550[3)]	0.634[2)]
**Total Protein (6.6~8.3g/dL)**	Visit 1	7.19±0.39	7.26±0.38	0.328[1)]					
	Visit 3	7.31±0.38	7.28±0.36	0.657[1)]	0.12±0.28	**0.002[3)]**	0.02±0.31	0.610[3)]	**0.048[2)]**
	Visit 4	7.34±0.39	7.37±0.42	0.609[1)]	0.14±0.29	**0.001[3)]**	0.11±0.32	**0.010[3)]**	0.578[1)]
**Albumin (3.5~5.2g/dL)**	Visit 1	4.37±0.22	4.39±0.21	0.830[2)]					
	Visit 3	4.36±0.21	4.36±0.20	0.944[2)]	-0.01±0.16	0.737[4)]	-0.02±0.17	0386[4)]	0.467[2)]
	Visit 4	4.35±0.21	4.38±0.22	0.417[2)]	-0.02±0.15	0.408[4)]	0.00±0.20	0.896[3)]	0.730[2)]
**AST (0~35U/L)**	Visit 1	23.37±7.64	26.98±13.03	0.139[2)]					
	Visit 3	23.87±7.54	26.58±11.62	0.225[2)]	0.50±7.58	0.385[4)]	-0.41±6.30	0.848[4)]	0.517[2)]
	Visit 4	23.13±5.92	26.76±11.36	0.071[2)]	-0.23±6.49	0.723[4)]	-0.22±7.55	0.859[4)]	0.928[2)]
**ALT (0~35U/L)**	Visit 1	19.08±9.80	20.58±10.52	0.326[2)]					
	Visit 3	20.35±10.35	20.25±8.99	0.709[2)]	1.27±10.62	0.384[4)]	-0.32±8.04	0.821[4)]	0.622[2)]
	Visit 4	19.02±8.01	21.08±9.46	0.223[2)]	-0.07±8.54	0.422[4)]	0.51±7.58	0.419[4)]	0.996[2)]
**ALP (30~120U/L)**	Visit 1	63.17±13.47	65.25±18.97	0.575[2)]					
	Visit 3	63.17±14.60	67.29±15.56	0.139[1)]	0.00±7.92	1.000[3)]	2.03±10.52	**0.048[4)]**	0.311[2)]
	Visit 4	64.45±13.88	69.64±17.11	0.072[1)]	1.28±8.06	0.222[3)]	4.39±11.89	**0.001[4)]**	0.258[2)]
**GGT (9~64U/L)**	Visit 1	18.75±11.61	21.93±23.56	0.907[2)]					
	Visit 3	19.12±12.32	21.32±19.03	0.600[2)]	0.37±7.21	0.677[4)]	-0.03±8.45	0.269[4)]	0.371[2)]
	Visit 4	19.47±12.78	22.78±20.78	0.704[2)]	0.72±7.19	0.061[4)]	0.85±12.27	0.098[4)]	0.856[2)]
**Total bilirubin (0.2~1.2mg/dL)**	Visit 1	0.77±0.24	0.78±0.27	0.652[2)]					
	Visit 3	0.79±0.29	0.80±0.29	0.991[2)]	0.02±0.25	0.656[4)]	0.02±0.22	0.748[4)]	0.953[4)]
	Visit 4	0.76±0.25	0.76±0.28	0.974[2)]	-0.01±0.22	0.589[4)]	-0.02±0.23	0.450[4)]	0.817[4)]
**CK (0~145U/L)**	Visit 1	94.13±54.80	167.75±344.12	**0.025[2)]**					
	Visit 3	104.75±67.38	116.92±72.69	0.246[2)]	10.62±65.95	**0.007[4)]**	-50.83±345.15	0.904[4)]	0.135[4)]
	Visit 4	95±46.76	109.75±61.40	0.180[2]	0.87±51.53	0.321[4)]	-58.00±346.26	0.843[4)]	0.449[4)]
**Triglyceride (Less than 150mg/dL)**	Visit 1	106.80±62.28	102.34±53.61	0.762[2)]					
	Visit 3	103.72±46.76	106.34±57.25	0.825[2)]	-3.8±54.33	0.431[4)]	400±36.70	0.432[4)]	0.962[4)]
	Visit 4	102.85±48.45	104.15±65.82	0.601[2)]	-3.95±61.25	0.803[4)]	1.81±56.47	0.704[4)]	0.657[4)]
**HDL (More than40mg/dL)**	Visit 1	57.53±12.74	59.75±13.78	0.356[1)]					
	Visit 3	58.20±12.63	60.24±13.26	0.393[1)]	0.67±6.26	0.413[3)]	0.49±4.97	0.451[3)]	0.866[1)]
	Visit 4	58.55±11.03	61.53±12.88	0.343[2)]	1.02±7.40	0.291[3)]	1.78±6.73	**0.045[4)]**	0.784[2)]
**LDL (0~130mg/dL)**	Visit 1	108.62±31.09	119.20±37.62	0.097[1)]					
	Visit 3	111.08±28.56	122.10±38.59	0.080[1)]	2.47±14.30	0.187[3)]	2.90±18.69	0.239[3)]	0.888[1)]
	Visit 4	110.88±29.42	118.83±37.32	0.200[1)]	2.27±23.51	0.458[3)]	-0.37±27.70	0.918[3)]	0.809[2)]
**hs-CRP (0~0.5mg/dL)**	Visit 1	0.08±0.13	0.09±0.10	0.133[2)]					
	Visit 3	0.10±0.19	0.09±0.14	0.156[2)]	0.03±0.15	0.133[4)]	0.00±0.10	0.968[4)]	0.392[2)]
	Visit 4	0.15±0.30	0.13±0.22	0.603[2)]	0.07±0.27	**0.047[4)]**	0.04±0.18	0.262[4)]	0.595[2)]
**Ca (8.2~10.5mg/dL)**	Visit 1	9.51±0.31	9.56±0.31	0.435[1)]					
	Visit 3	9.53±0.33	9.54±0.28	0.827[1)]	0.01±0.25	0.677[3)]	-0.02±0.25	0.568[3)]	0.483[1)]
	Visit 4	9.52±0.31	9.53±0.33	0.837[1)]	0.00±0.25	0.920[3)]	-0.03±0.29	0.453[3)]	0.524[1)]
**P (2.5~4.5mg/dL)**	Visit 1	3.70±0.52	3.76±0.55	0.585[2)]					
	Visit 3	3.70±0.51	3.79±0.56	0.317[1)]	0.00±0.39	0.950[4)]	0.03±0.40	0.518[3)]	0.607[1)]
	Visit 4	3.70±0.49	3.77±0.51	0.415[1)]	0.00±0.41	0.745[4)]	0.01±0.51	0.859[3)]	0.470[2)]
**Na (136~146mg/dL)**	Visit 1	139.32±1.92	139.97±1.88	0.142[2)]					
	Visit 3	139.20±1.55	139.37±1.71	0.546[2)]	-0.12±1.5	0.607[4)]	-0.59±1.33	0.002[4)]	0.051[2)]
	Visit 4	139.72±1.69	156.83±129.95	0.503[2)]	0.40±1.75	0.060[4)]	16.86±130.34	0.959[4)]	0.184[2)]
**K (3.5~5.1mg/dL)**	Visit 1	4.13±0.27	4.24±0.35	0.158[2)]					
	Visit 3	4.27±0.36	4.39±0.39	**0.041[2)]**	0.14±0.31	**0.001[4)]**	0.16±0.35	**0.001[4)]**	0.932[2)]
	Visit 4	4.21±0.27	4.27±0.29	0.251[1)]	0.08±0.24	**0.014[3)]**	0.03±0.33	**0.238[4)]**	0.596[2)]
**Cl (101~109mmol/L)**	Visit 1	104.97±1.59	104.88±2.08	0.637[2)]					
	Visit 3	104.70±1.70	104.61±1.55	0.812[2)]	-0.27±1.81	0.256[4)]	-0.27±1.63	0.256[4)]	0.875[2)]
	Visit 4	104.78±1.47	104.81±2.15	0.940[2)]	-0.18±1.33	0.213[4)]	-0.07±1.72	0.563[4)]	0.597[2)]

Data are reported as mean ± standard deviation. The Shapiro–Wilk test was applied to assess data normality. * *P* values indicate comparisons within groups. ** *P* values indicate comparisons between groups. Statistical analyses: 1) independent *t*-test, 2) Mann–Whitney U test, 3) paired *t*-test, 4) Wilcoxon's signed rank test.

Table S9. Comparison of vital signs (body temperature, blood pressure, pulse rate) within and between groups in the ITT population.

Variable	Observed value			Difference from baseline				
	Control (n=60)	Experimental (n=59)	*p*-value**	Control	*p*-value*	Experimental	*p*-value*	*p*-value**
**Body temperature**								
visit 1	36.52±0.13	36.48±0.08	0.101[2)]					
visit 3	36.37±0.12	36.39±0.14	0.334[2)]	-0.15±0.19	**<0.001[4)]**	-0.09±0.17	**<0.001[4)]**	0.091[2)]
visit 4	36.41±0.12	36.38±0.12	0.258[2)]	-0.11±0.18	**<0.001[4)]**	-0.10±0.14	**<0.001[4)]**	0.879[2)]
**Activity level**								
visit 1	75.65±9.30	75.14±9.90	0.785[1)]					
visit 3	75.85±10.22	75.03±10.24	0.529[1)]	-1.77±8.61	0.117[3)]	-0.10±7.54	0.918[3)]	0.264[1)]
visit 4	75.08±10.46	74.76±11.27	0.872[1)]	-0.53±7.12	0.564[3)]	-0.37±8.81	0.746[3)]	0.913[1)]
**Systolic blood pressure**								
visit 1	128.08±14.41	127.97±14.13	0.964[1)]					
visit 3	124.53±13.53	126.76±14.13	0.381[1)]	-3.55±12.25	**0.0.29[3)]**	-1.20±12.15	0.450[3)]	0.296[1)]
visit 4	126.57±15.01	126.39±14.74	0.948[1)]	-1.52±10.30	0.0.29[3)]	-1.58±10.90	0.271[3)]	0.890[2)]
**Pulse rate**								
visit 1	75.07±8.47	75.81±8.77	0.637[1)]					
visit 3	74.83±10.24	75.17±9.79	0.647[2)]	-0.23±9.66	**0.331[4)]**	-0.64±8.63	0.569[3)]	0.909[2)]
visit 4	74.23±9.73	74.41±10.08	0.924[1)]	-0.83±6.93	0.355[3)]	-1.41±9.20	0.245[3)]	0.827[2)]

Data are reported as mean ± standard deviation. The Shapiro–Wilk test was used to evaluate the assumption of normality. * *P* values represent within-group comparisons. ** ***P*** values represent between-group comparisons. Statistical analyses: 1) independent *t*-test, 2) Mann–Whitney U test, 3) paired *t*-test, 4) Wilcoxon's signed rank test.

Table S10. Comparison of medical history obtained from questionnaires in the ITT population.

Variable		Visit 1(=Visit 2)			Visit 3			Visit 4		
		Control (n=60)	Experimental (n=59)	*p*-value**	Control (n=60)	Experimental (n=59)	*p*-value**	Control (n=60)	Experimental (n=59)	*p*-value**
**Nutritional status**	**Normal**	60 (100%)	59 (100%)	-	57 (100%)	59 (100%)	-	55 (100%)	58 (100%)	-
	**Abnormal**	0 (0.0%)	0 (0.0%)		0 (0.0%)	0 (0.0%)		0 (0.0%)	0 (0.0%)	
**Respiratory system**	**Normal**	60 (100%)	59 (100%)	-	57 (100%)	59 (100%)	-	55 (100%)	58 (100%)	-
	**Abnormal**	0 (0.0%)	0 (0.0%)		0 (0.0%)	0 (0.0%)		0 (0.0%)	0 (0.0%)	
**Skin/Hair**	**Normal**	60 (100%)	59 (100%)	-	57 (100%)	59 (100%)	-	55 (100%)	58 (100%)	-
	**Abnormal**	0 (0.0%)	0 (0.0%)		0 (0.0%)	0 (0.0%)		0 (0.0%)	0 (0.0%)	
**Metabolic/Endocrine system**	**Normal**	60 (100%)	59 (100%)	-	57 (100%)	59 (100%)	-	55 (100%)	58 (100%)	-
	**Abnormal**	0 (0.0%)	0 (0.0%)		0 (0.0%)	0 (0.0%)		0 (0.0%)	0 (0.0%)	
**Otorhinolaryngology**	**Normal**	60 (100%)	59 (100%)	-	57 (100%)	59 (100%)	-	55 (100%)	58 (100%)	-
	**Abnormal**	0 (0.0%)	0 (0.0%)		0 (0.0%)	0 (0.0%)		0 (0.0%)	0 (0.0%)	
**Digestive system**	**Normal**	60 (100%)	59 (100%)	-	57 (100%)	59 (100%)	-	55 (100%)	58 (100%)	-
	**Abnormal**	0 (0.0%)	0 (0.0%)		0 (0.0%)	0 (0.0%)		0 (0.0%)	0 (0.0%)	
**Cardiovascular system**	**Normal**	60 (100%)	59 (100%)	-	57 (100%)	59 (100%)	-	55 (100%)	58 (100%)	-
	**Abnormal**	0 (0.0%)	0 (0.0%)		0 (0.0%)	0 (0.0%)		0 (0.0%)	0 (0.0%)	
**Musculoskeletal system**	**Normal**	60 (100%)	59 (100%)	-	57 (100%)	59 (100%)	-	55 (100%)	58 (100%)	-
	**Abnormal**	0 (0.0%)	0 (0.0%)		0 (0.0%)	0 (0.0%)		0 (0.0%)	0 (0.0%)	
**Renal/Urogenital system**	**Normal**	60 (100%)	59 (100%)	-	57 (100%)	59 (100%)	-	55 (100%)	58 (100%)	-
	**Abnormal**	0 (0.0%)	0 (0.0%)		0 (0.0%)	0 (0.0%)		0 (0.0%)	0 (0.0%)	
**Neurological/Psychiatric system**	**Normal**	60 (100%)	59 (100%)	-	57 (100%)	59 (100%)	-	55 (100%)	58 (100%)	-
	**Abnormal**	0 (0.0%)	0 (0.0%)		0 (0.0%)	0 (0.0%)		0 (0.0%)	0 (0.0%)	
**Other systems**	**Normal**	60 (100%)	59 (100%)	-	57 (100%)	59 (100%)	-	55 (100%)	58 (100%)	-
	**Abnormal**	0 (0.0%)	0 (0.0%)		0 (0.0%)	0 (0.0%)		0 (0.0%)	0 (0.0%)	

Data are presented as frequency (percentage). *P* values were calculated using 1) the Chi-square test or 2) Fisher's exact test.

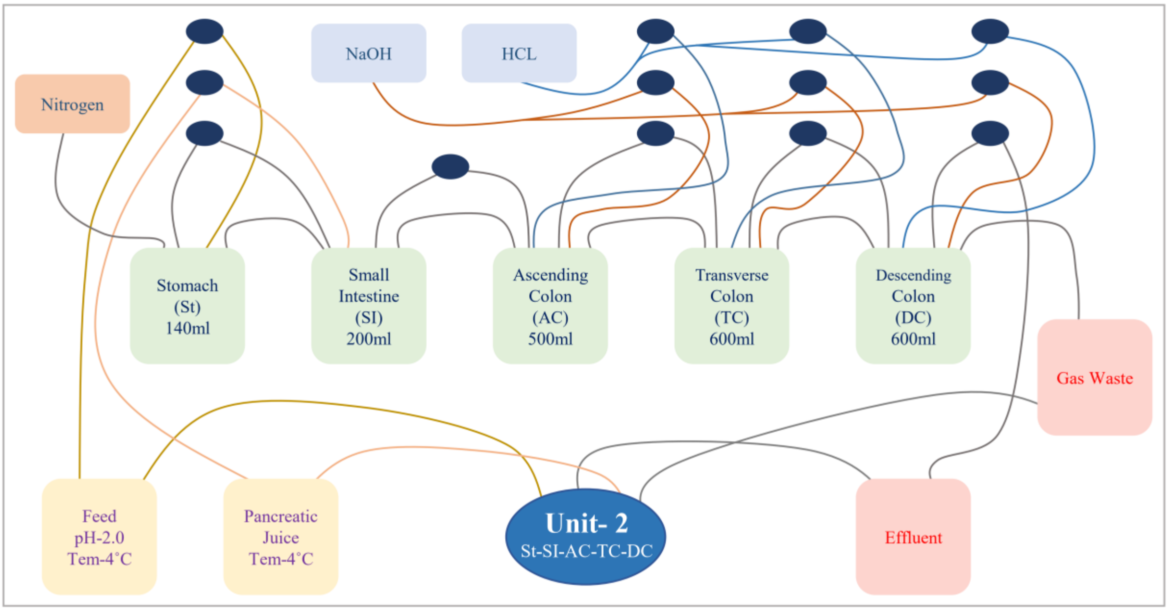

Fig. S1. A schematic diagram of human gut microbiome simulation systems. This advanced in vitro model simulates the human gastrointestinal tract, including the stomach (S), small intestine (SI), and three colon compartments: the ascending colon (AC), transverse colon (TC), and descending colon (DC). This diagram highlights the flow of materials and treatments across different compartments. The color-coded lines represent the transfer pathways.

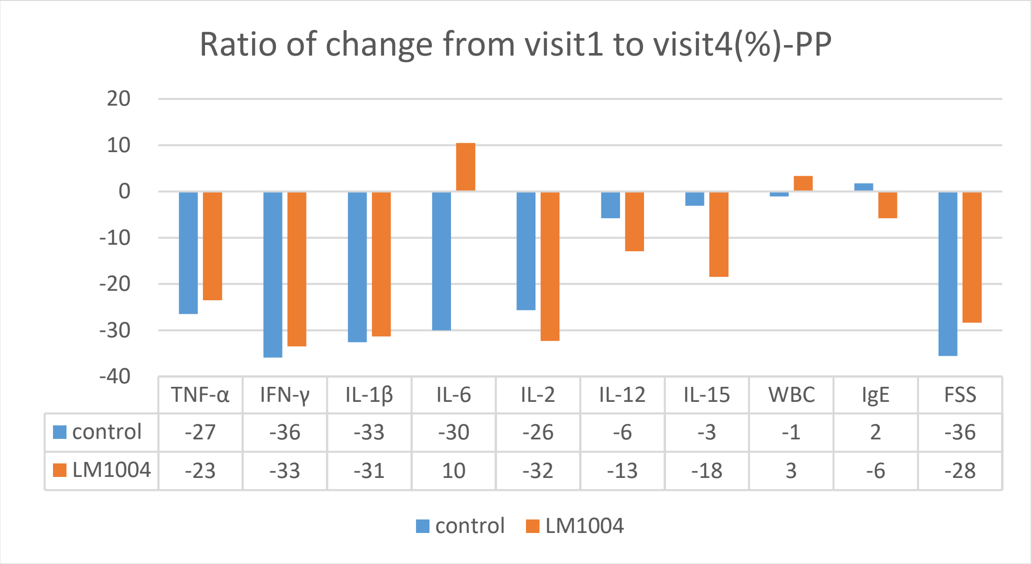

Fig. S2. Percentage change in inflammatory markers and immune parameters from Visit 1 to Visit 4 (Per-Protocol analysis). Bar graphs show the relative percentage change from baseline (Visit 1) to the end of intervention (Visit 4) in the control and LM1004 groups. Parameters include pro- and anti-inflammatory cytokines (TNF-α, IFN-γ, IL-1β, IL-6, IL-2, IL-12, IL-15), white blood cell count (WBC), immunoglobulin E (IgE), and fatigue severity score (FSS). Values represent the percentage change from baseline within each group.

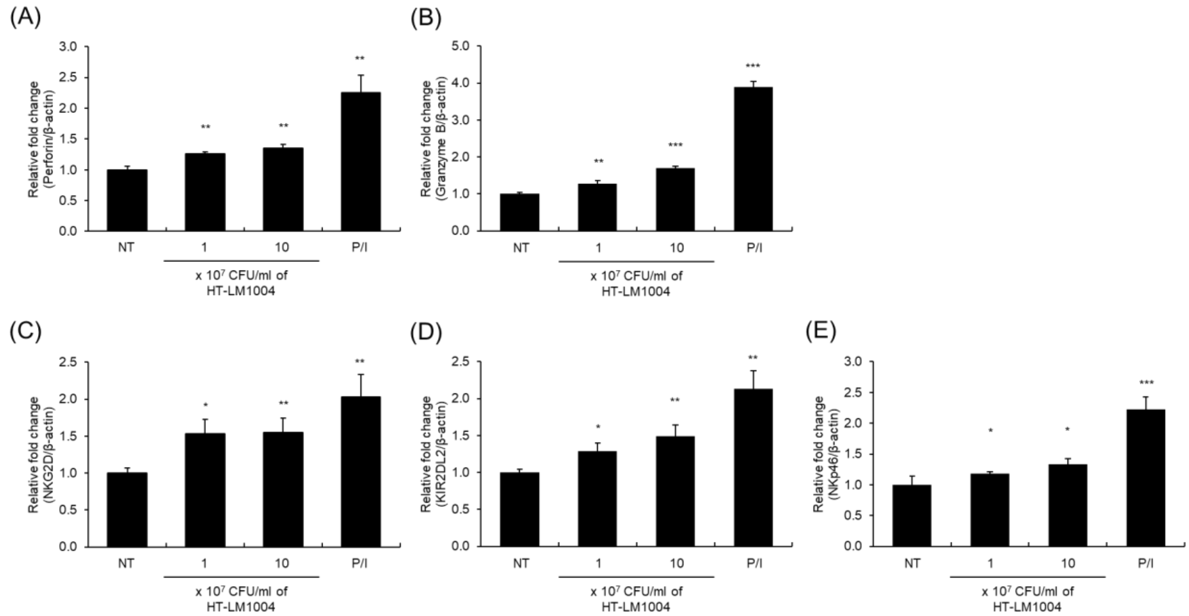

Fig. S3. HT-LM1004 upregulates gene expression of cytotoxic NK cell-associated receptor in NK3.3 cells. NK3.3 cells were seeded in a 24-well plate and stimulated with the indicated concentrations of HT-LM1004 and PMA/Ionomycin (P/I) for 24 h. Total RNA was isolated, and the mRNA expression of **(A)** perforin, **(B)** granzyme B, **(C)** NKG2D, **(D)** KIR2DL2, and **(E)** NKp46 was analyzed using real-time RT-PCR. All results were expressed as mean ± standard deviation (S.D.) of triplicate samples. Asterisk (*) indicates statistically significant differences ($P < 0.05$) compared with the appropriate control. NT, non-treatment.
